# Hypochlorous Acid Chemistry in Mammalian Cells—Influence on Infection and Role in Various Pathologies

**DOI:** 10.3390/ijms231810735

**Published:** 2022-09-14

**Authors:** Celia María Curieses Andrés, José Manuel Pérez de la Lastra, Celia Andrés Juan, Francisco J. Plou, Eduardo Pérez-Lebeña

**Affiliations:** 1 Hospital Clínico Universitario de Valladolid, Avenida de Ramón y Cajal, 3, 47003 Valladolid, Spain; 2 Institute of Natural Products and Agrobiology, IPNA-CSIC-Spanish Research Council, Avda. Astrofísico Fco. Sánchez, 38206 San Cristóbal de La Laguna, Spain; 3 Cinquima Institute and Department of Organic Chemistry, Faculty of Sciences, Valladolid University, Paseo de Belén, 7, 47011 Valladolid, Spain; 4 Institute of Catalysis and Petrochemistry, CSIC-Spanish Research Council, 28049 Madrid, Spain; 5 Sistemas de Biotecnología y Recursos Naturales, 47625 Valladolid, Spain

**Keywords:** hypochlorous acid HOCl, halogenative stress, myeloperoxidase MPO, pathologies

## Abstract

This review discusses the formation of hypochlorous acid HOCl and the role of reactive chlorinated species (RCS), which are catalysed by the enzyme myeloperoxidase MPO, mainly located in leukocytes and which in turn contribute to cellular oxidative stress. The reactions of RCS with various organic molecules such as amines, amino acids, proteins, lipids, carbohydrates, nucleic acids, and DNA are described, and an attempt is made to explain the chemical mechanisms of the formation of the various chlorinated derivatives and the data available so far on the effects of MPO, RCS and halogenative stress. Their presence in numerous pathologies such as atherosclerosis, arthritis, neurological and renal diseases, diabetes, and obesity is reviewed and were found to be a feature of debilitating diseases.

## 1. Introduction

Bacteria are constantly confronted with environmental stressors, such as pH and temperature extremes, osmotic pressure, antimicrobial agents, and oxidants, which can induce irreversible damage. Among the most widespread reactive agents are reactive oxygen species (ROS), such as superoxide anion, hydrogen peroxide and hydroxyl radical, as well as reactive chlorine species (RCS), such as hypochlorous acid [1]. Neutrophils destroy pathogenic invaders by ingesting them through phagocytosis and by releasing antimicrobial proteins, oxidants, and digestive enzymes. In addition, stimulated neutrophils also participate in the respiratory burst, which is accompanied by an increase in oxygen consumption and the consequent production of ROS by the enzyme NADPH-oxidase. This enzyme complex assembles at the plasma membrane and transfers electrons from NADPH to molecular oxygen, forming the superoxide anion ^•^O_2_^−^. Then, the enzyme superoxide dismutase (SOD) converts ^•^O_2_^−^ into hydrogen peroxide H_2_O_2_, a precursor of several toxic species [2] and, on the other hand, according to Winterbourn et al., 2006, MPO converts ^•^O_2_^−^ into hydrogen peroxide H_2_O_2_ [2]. In the cell, there are different defence mechanisms to prevent damage induced by these species, including antioxidants (such as ascorbic acid and β-carotene), and enzymes (catalase, superoxide dismutase, and glutathione peroxidase). However, under conditions of oxidative stress, due to the overproduction of oxidative species, or when antioxidant levels are severely depleted, these species become detrimental, causing the oxidation of biomolecules, and may ultimately lead to cell death [3].

**Figure 1:**
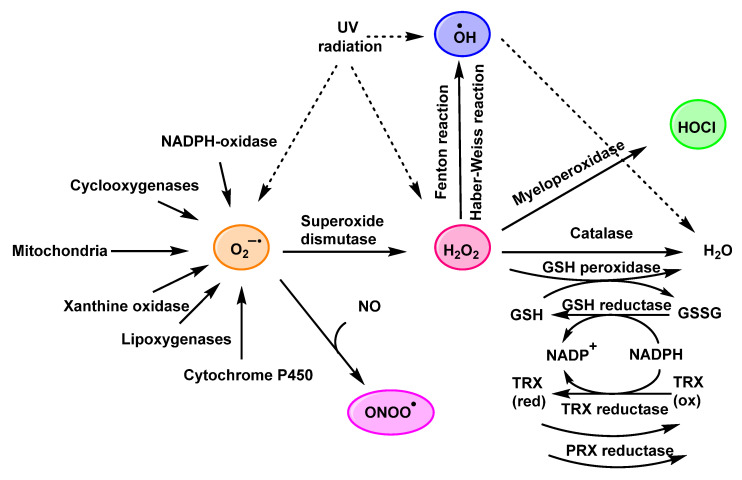
Chemical and enzymatic reactions generating ROS and main antioxidant systems.

In the 1970s and 1980s, the term “oxidative stress” was used to describe this process. Later, the German biochemist Helmut Sies defined it as an imbalance between oxidants and antioxidants in favour of oxidants [4]. The formation of the different reactive species is shown in Figure 1.

## 2. Enzyme Myeloperoxidase (MPO) and Its Catalytic Cycle

Myeloperoxidase (MPO), also called hydrogen peroxide oxidoreductase with a particular (EC 1.11.1.7), is an enzyme found in the primary granules of granulocytic cells (neutrophils, eosinophils, and, to a lesser extent, monocytes). Lymphocytes lack MPO enzyme activity. However, the most common sources are neutrophils, where the enzyme is located at the lysosomal level in the azurophil granules [5].

MPO is the major component of white blood cells in humans [6]. These leukocytes recognise microorganisms through various receptors that act by stimulating the migration of the cells to the site of infection, promoting the phagocytosis of the microorganisms and stimulating the production of biocidal substances that destroy the microorganisms. A second microbicidal mechanism used by activated leukocytes occurs during the respiratory burst, which involves reducing molecular oxygen to reactive oxygen intermediates (ROS), such as superoxide radicals ^•^O_2_^−^, using the reduced form of NADPH [7]. The superoxide undergoes a disproportionate reaction to give oxygen (O_2_) and hydrogen peroxide (H_2_O_2_), and the latter is used by the MPO enzyme to convert the normally very unreactive halide ions into hypohalous acids that are relatively strong oxidising agents and toxic to bacteria [8]. When intense leukocyte activation occurs, ROS, nitric oxide, and lysosomal enzymes are released, which can injure normal host tissues [9].

**Figure 2:**
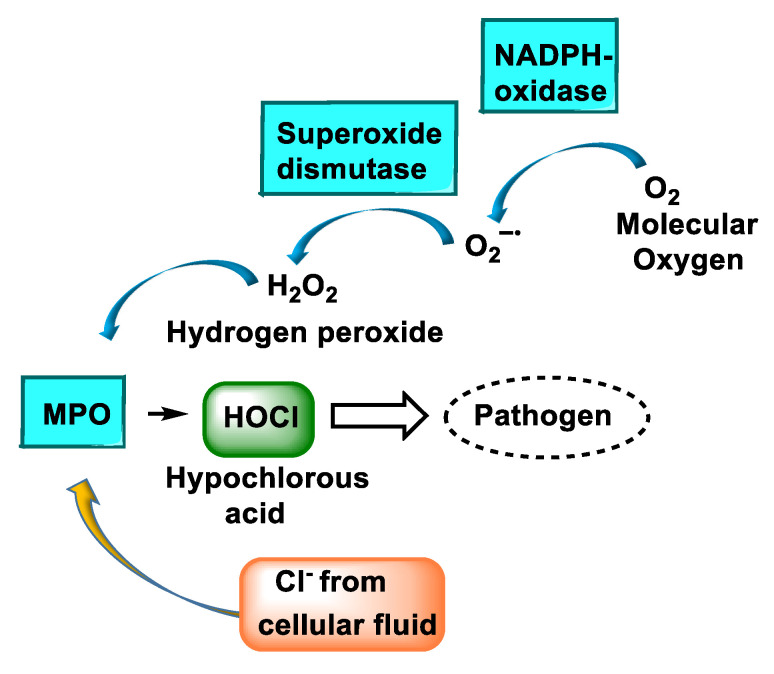
Schematic representation of HOCl production during the respiratory burst. The cell uses molecular oxygen (O_2_) to produce H_2_O_2_, using the enzyme NADPH oxidase. MPO deposited on the azurophilic granules catalyses the reaction of H_2_O_2_ and the chloride anion Cl^−^ to form hypochlorous acid HOCl.

The main reaction catalysed by MPO, under physiological conditions, is the oxidation of the Cl^−^ anion by H_2_O_2_ to give hypochlorous acid HOCl, a very reactive oxidising agent, which can also act as a chlorinating agent [8,10,11,12,13], Figure 2.

Myeloperoxidase plays a key role in the production of oxidants by neutrophils. This heme-enzyme uses hydrogen peroxide and chloride to catalyse the production of hypochlorous acid, the main strong oxidant generated by neutrophils in appreciable quantities. In addition to chlorination, myeloperoxidase exhibits other activities. It readily oxidises thiocyanate to hypothiocyanite, converts a variety of organic substrates to reactive free radicals, and hydroxylates aromatic compounds. Depending on the concentration of its competing substrates and local environmental conditions, myeloperoxidase may substantially affect the production of oxidants by neutrophils. MPO is the only peroxidase that catalyses the conversion of H_2_O_2_ and chloride to produce hypochlorous acid (HOCl) [14]. This is a potent oxidising agent that contributes to the defence mechanism against infectious agents; however, it may be capable of acting on the body’s own cells in the case of prolonged and extended activation of this enzyme. In these cases, the high oxidative power of HOCl is involved in the mismatch of the redox state and can consequently lead to systemic oxidative stress. The MPO system is also involved in leukocyte functions such as the chemical modifications of plasma and tissue components, the elimination of abnormal host cells and inflammatory processes [15].

**Figure 3:**
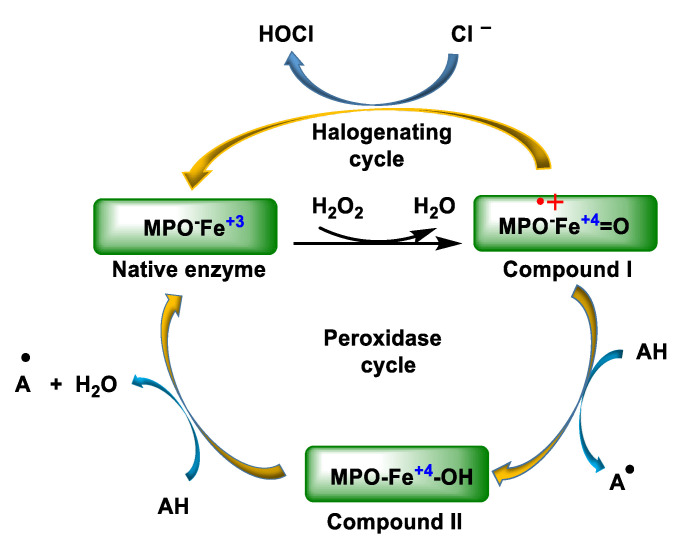
Normal catalytic cycling of myeloperoxidase. The native state of the enzyme, ferric MPO, reacts with hydrogen peroxide to form the redox intermediate Compound I. Compound I oxidises chloride to regenerate ferric MPO via the halogenation cycle.

The native enzyme MPO is a protein with a central iron in the heme and MPO-Fe^3+^ can be at various oxidation states, as shown in Figure 3. MPO-Fe^+3^ reacts rapidly and irreversibly with hydrogen peroxide from phagocytic cells activated by contact with foreign particles, forming an enzyme–substrate complex with a strong oxidative capacity. Compound I can be reduced to compound II by an excess of H_2_O_2_ or by the presence of a reducing agent. This compound II, although quite stable, can be reduced to ferric MPO in the presence of a reducing agent or the superoxide radical or it can also be oxidised to compound III under high concentrations of H_2_O_2_. On the other hand, native MPO reacts with superoxide to give compound III.

The MPO enzyme uses H_2_O_2_ to oxidise chloride, but it also oxidises bromide Br^−^, iodide I^−^, and thiocyanate SCN^−^, taking them to their respective hypohalogenated acids. At neutral pH values, physiological plasma ion concentrations are ~100 mM Cl^−^, 20–100 µM Br^−^, <1 µM I^−^, 20–100 µM SCN^−^ [16], and MPO primarily generates HOCl and HOSCN (which can be a significant product due to its rapid oxidation by Compound I), and significant levels of this anion are found in smokers and in vegetarians and vegans who consume diets rich in plants and fruits [17]. HOI formation is usually insignificant, although it is generated rapidly, due to the low plasma levels of I^−^, varying significantly with diet (lower levels are detected in vegans than vegetarians). HOBr is usually formed at low concentrations, it is pH dependent, and can also be improved by means of food supplements [18].

The relative specificity constants for chloride, bromide and thiocyanate are 1:60:730, respectively, with thiocyanate being the most favoured substrate for MPO. In the presence of 100 mM Cl^−^, MPO catalyses the hypothiocyanite production at thiocyanate concentrations of 25 µM. At 100 µM thiocyanate, roughly 50% of peroxide is transformed into hypothiocyanite, and the quantity of hypohalogenic acid production was equal to the sum of the individual rates obtained when each of these negative ions was present alone. The percentage of MPO-induced H_2_O_2_ loss in the presence of 100 mM Cl^−^ doubled when 100 µM thiocyanate was added and was maximal at 1 mM thiocyanate. This indicates that at plasma concentrations of thiocyanate and chloride, myeloperoxidase is far from saturated. Thus, thiocyanate is an important physiological substrate of MPO [19].

**Figure 4:**
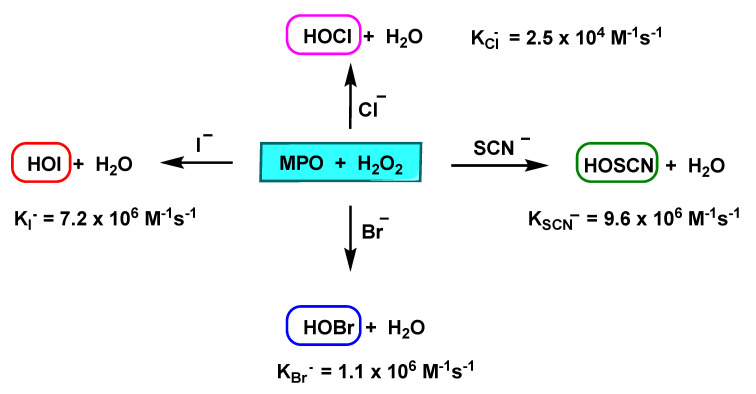
Catalysed conversion of Cl^−^, Br^−^, I^−^, and SCN^−^ by MPO, with second-order constants K.

In general, SCN^−^ is not considered a biologically functional ion, however, it plays an important role as a substrate for peroxidase enzymes, which form an essential part of innate defence [20]. Peroxidases catalyse the oxidation of halides (Cl^−^, Br^−^, I^−^) and pseudohalides (SCN^−^) by H_2_O_2_, resulting in potent oxidising agents that exhibit antimicrobial activity in vitro and in vivo, as they damage the vital structural and functional components of microorganisms. They oxidised acceptor molecules, e.g., bromide Br^−^ to hypobromite BrO^−^, iodide I^−^ to hypoiodite IO^−^, and thiocyanate SCN^−^ to hypothiocyanite OSCN^−^, as shown in Figure 4.

MPO oxidises chloride, bromide, iodide, and thiocyanate ions (H=Cl^−^, Br^−^, I^−^, and SCN^−^), to the respective HOX acids. With a 100 mM concentration of Cl^−^, MPO catalyses OSCN^−^ production at SCN^−^ concentrations of 25 µM, and at 100 µM of SCN^−^, approximately 50% of H_2_O_2_ is converted to OSCN^−^ [19]. The loss of H_2_O_2_ induced by MPO in the presence of 100 mM Cl^−^ doubled when 100 µM of SCN^−^ was added and was maximal at 1 mM of SCN^−^. It is concluded that SCN^−^ is an important physiological substrate of MPO [19]. The transition from SCN^−^ to HOSCN by MPO is an important detoxification mechanism by removing the oxidant H_2_O_2_ and subsequently HOCl [21].

The pKa values of the resulting hypohalous acids are: 7.59 for HOCl, 8.7 for HOBr, 10.4 for HOI, and 4.85 for HOSCN [22]. The hypohalous acids HOX are in equilibrium with their anions OX^−^. At physiological pH values, OSCN^−^ predominates over HOSCN, HOBr over OBr^−^ and HOI over OI^−^, in nearly equal mixtures of HOCl and OCl^−^.

The MPO enzyme plays an important defensive role in innate immunity, as the HOX acids it produces facilitate the destruction of invading bacteria and pathogens. HOCl is the main strong oxidant generated by neutrophils and a powerful microbicidal mediator. In vitro, one million stimulated neutrophils can produce 0.1 µM of HOCl and this concentration can kill 15 million *Escherichia coli* bacteria in less than 5 min [23]. HOCl reacts quickly with some biological molecules, mostly those with thiols, thioethers, heme proteins, and amino groups, triggering tissue injury [24]. Taurine, at a concentration of 15 mM, is found naturally in cells and acts as a scavenger of HOCl, safeguarding collateral damage to cellular macromolecules (proteins, lipids, and DNA). Taurine present in human plasma and cells and HOCl form taurine chloramine (–O_3_SCH_2_CH_2_NHCl), a weaker oxidant with greater stability [25]. Tau-NHCl accumulates in the extracellular medium and does not inhibit neutrophil functions, may moderate neutrophil cytotoxic activity long after HOCI has been eliminated.

Endogenic hypohalous acids can also provoke tissue damage at the sites of inflammation, an area of research in neurodegenerative disorders, Alzheimer’s and Parkinson’s diseases, metabolic and cardiovascular dysfunction (atherosclerosis, diabetes), autoimmune dysregulation and ageing, among other conditions. MPO is associated with various skin pathologies: such as hypersensitivity and contact irritation, psoriasis, UV damage, photoaging or melanoma [26].

## 3. Formation and Features of Hypochlorous Acid HOCl

**Figure 5:**
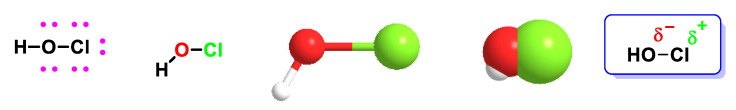
In hypochlorous acid, the chlorine–oxygen bond is polarised as shown in the figure, so Cl^+^ will act as an electrophilic reactant.

Biologically, HOCl is classified within a group of molecules known as reactive oxygen species (ROS) synthesised by cells of the immune system (neutrophils and macrophages). This agent can participate in subsequent non-enzymatic reactions such as the oxidation and chlorination of cellular components and can also react with substances present in the environment that modulate its biological effects [15], as shown in Figure 5.

The usual behaviour of hypochlorous acid with the carbon and nitrogen of the amino group is that of an electrophilic agent, in which the chlorine atom combines with a pair of electrons from the substrate, while the hydroxide ion is simultaneously or subsequently separated, often assisted by a proton from the solvent or from a reactive centre elsewhere in the substrate. This feature mainly occurs in reactions with ammonia nitrogen, amides, amino acids, and peptides, with phenolic and other aromatic compounds. Cl^+^ transfer is favoured by the negative charge, basicity, and nucleophilicity of the receiving acid [27].

Although H_2_O_2_ is a strong oxidising agent, it does not readily react with biological molecules in the absence of catalysts. In addition, leukocytes and most cells possess enzymes that rapidly remove H_2_O_2_ by converting it into O_2_ and/or H_2_O. In contrast, HOCl and chloramines react rapidly with biological molecules, and no specific enzymatic mechanism is known to remove these oxidants.

**Figure 6:**
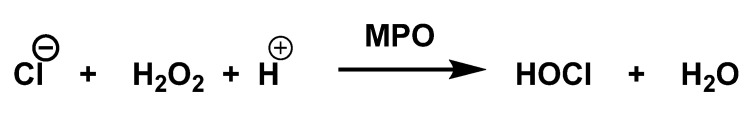
Production of hypochlorous acid from hydrogen peroxide and chloride.

Hypochlorous acid is the main strong oxidant generated by neutrophils. The enzyme MPO catalyses the production of hypochlorous acid from hydrogen peroxide and chloride [28], as shown in Figure 6.

HOCl and the associated hypochlorite anion are present in the same concentrations, because the pKa of HOCl is ~7.5 in aqueous media at physiological pH values [29,30].

Approximately 70% of the H_2_O_2_ produced in neutrophils is converted into HOCl [31]. Excessive HOCl generation can cause tissue damage and is thought to be important in the progression of several diseases including atherosclerosis, chronic inflammation, and some cancers. When the human body is invaded by bacteria or viruses, our immune system responds immediately by sending an increased number of white blood cells called neutrophils to the site of invasion. Once activated, neutrophils produce large amounts of HOCl through the enzyme myeloperoxidase, which is effective in killing all pathogenic invading microbes [1].

HOCl is considered an extremely powerful oxidant. It is estimated that 106 stimulated neutrophils can produce approximately 0.2 µmol of HOCl during 2 h of incubation [1]. In a matter of milliseconds, 0.2 µmol of HOCl is sufficient to destroy 150 million *E. coli* cells [32]. HOCl is involved in the destruction of bacterial cells [32], penetrating through cell walls with a water-like ease, mainly due to its low molecular weight and its electroneutrality [33]. Once inside the cell, thanks to its strong oxidising power, it can react with a large number of biological molecules, especially DNA, RNA, thiols, heme-proteins, amino groups, carbohydrates, and lipids [34].

Although HOCl has potent bactericidal properties and plays an important role in the human immune system, this oxidant also causes tissue damage, particularly under inflammatory conditions. There is a strong link between chronic inflammation and the incidence of many types of cancer, which may be associated with the ability of HOCl and related oxidants, such as *N*-chloramines, to damage DNA [35].

HOCl is part of a new group of microbicidal substances known as “non-antibiotic antimicrobial molecules” which, due to their broad spectrum, fast action, wide safety margin, concentration, and form of stabilisation, can be used in disinfection processes and/or to control and prevent a wide range of skin and mucosal infections [36].

HOCl plays a powerful microbicidal role in the innate immune system. The regulated production of microbicidal HOCl is required for the host to control invading microorganisms. However, due to the highly reactive nature and diffusibility of HOCl, its uncontrolled production may trigger undesirable consequences on mammal physiology [37]. For HOCl, the pKa value is 7.59 and at physiological pH, there is nearly equal mixtures of HOCl and OCl^−^. After formation, the ClO^−^ diffuses away the enzyme cavity, and it is rapidly protonated according to ClO^−^/HOCl equilibrium.

The harmful actions induced by these species persist for long periods, even after the cessation of its production. Possibly, much of the secondary damage arises from the reaction of long-lived chloramines/chloramides and bromamines/bromamides, through HOCl/HOBr reaction with amines and amides. The longer lifetimes of these species allow their diffusion out of the site of formation, across cell membranes, unleashing damage in remote places. These species, which are generated extracellularly, can exert intracellular effects, and the degree of penetration into the cell is dependent on the structure of the halogenated species [8].

## 4. HOCl Reaction with Lipids

Phospholipids, fatty acids, sterols, and sphingolipids are susceptible to HOCl oxidation. Alkenes in the aliphatic residues of esterified and non-esterified fatty acids as well as the double bond in cholesterol are oxidised to chlorohydrins. Another lipid target of HOCl is the primary amino groups present in ethanolamine and serine glycerophospholipids. The main targets of reactive chlorine species RCS in LDL lipids are unsaturated –CH=CH– bonds of fatty acids and cholesterol and NH_2_ groups of polar heads of some phospholipids [38].

### 4.1. HOCl Reaction with Unsaturated Bonds

Chlorohydrins are formed by adding HOCl to the double bonds of unsaturated fatty acids and cholesterol, and these can be readily detected by gas chromatography–mass spec GC-MS [39]. These products can have physiological effects, including destabilisation of membrane structures and cytotoxicity, but the rate of formation of these materials is relatively slow and they are therefore relatively minor products in most situations [40].

Hypochlorous acid reacts with unsaturated bonds in lipids, but not with saturated bonds, and the ClO^−^ ion does not participate in this reaction. It is an electrophilic addition reaction of a strongly polarised asymmetric reactant, HO–Cl, to alkenes which may be asymmetrically substituted, in this case, giving two isomeric products. The formation of chlorohydrin results from the electrophilic attack of the pi bond to give an intermediate chloronium ion. The opening of this cyclic ion by the dorsal attack of the hydroxyl or water gives as the final product the addition of chlorine to one of the carbons and a hydroxyl to the other carbon. The resulting compound is a chlorohydrin.

**Figure 7:**
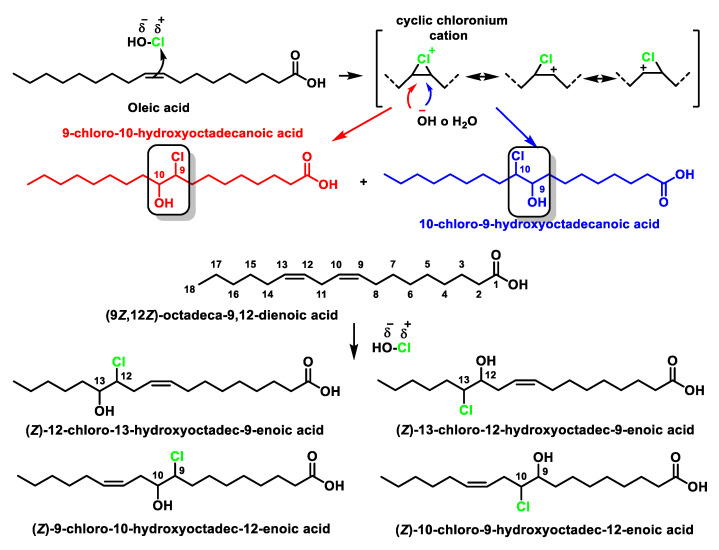
Electrophilic addition of HOCl to unsaturated fatty acids.

Oleic acid, an 18-carbon unsaturated fatty acid, adds chlorine to the C=C double bond to rapidly form two isomeric chlorohydrins, as shown in Figure 7.

If a fatty acid chain contains several unsaturated bonds, any of them can be attacked by HOCl. In the presence of excess hypochlorous acids, bis-chlorohydrins and monochlorohydrins [41] are formed on all the double bonds of the acids [42]. This gives rise to a complex pattern of positioning and stereoisomers, which can be characterised by their fragmentation patterns in mass spectrometry and by nuclear magnetic resonance NMR. Chlorohydrins by HCl elimination form epoxides under slightly alkaline conditions, but otherwise chlorohydrins are not highly reactive compounds [42].

Cholesterol is one of the most abundant steroids, present in almost all animal tissues, especially in the brain and spinal cord. The human body contains 200–300 g of cholesterol. In cholesterol, the sole target of hypochlorous acid is the double bond at the 5, 6-position of the steroid nucleus mainly to lead to the formation of the α- and β-isomers of chlorohydrins [43]. The same products are formed in LDL treated with HOCl or in the presence of MPO/H_2_O_2_/Cl^−^ [44,45].

Studies of hypercholesterolaemia and hypertriglyceridemia indicate that elevated levels of low-density lipoproteins LDL are the main cause of cardiovascular disease. Apolipoprotein B is part of lipoproteins that are considered atherogenic and promoters that develop atherosclerosis. There are two forms of apolipoprotein B100 (ApoB100) and B48 (ApoB48). ApoB100 is present in very low-density lipoproteins (VLDL), intermediate density lipoproteins IDL, and low-density lipoproteins LDL.

**Figure 8:**
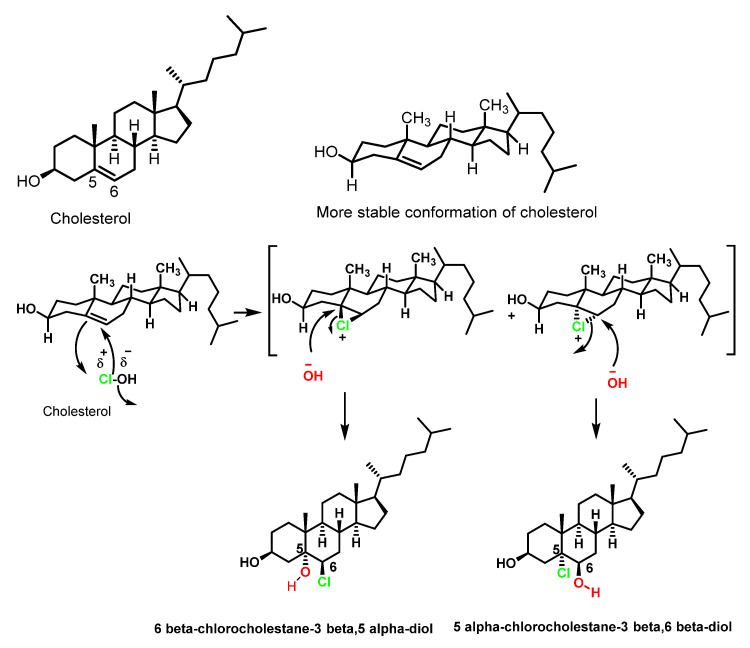
Electrophilic addition of HOCl to cholesterol. Mechanism of formation of cholesterol chlorohydrins.

The products were identified by gas chromatography–mass spectrometry as cholesterol α- and β-chlorohydrins (6β-cholesterol-3β, 5α-diol and 5α-cholesterol-3β, 6β-diol), cholesterol α- and β-epoxides (cholesterol 5α, 6β-epoxide and cholesterol 5β, 6β-epoxide), and a new cholesterol hydrochloride. The conversion of cholesterol into the oxidation products required active myeloperoxidase, hydrogen peroxide, and halide, and could be blocked by catalase or by HOCl scavengers [46]. Furthermore, in the absence of the enzyme system, the HOCl reagent generated the same product distribution. These results indicate that myeloperoxidase can convert cholesterol into chlorohydrins and epoxides via a reaction involving HOCl. Other oxygenated sterols are cytotoxic and mutagenic and are potent regulators of cholesterol homeostasis in cultured mammalian cells [47], Figure 8.

**Figure 9:**
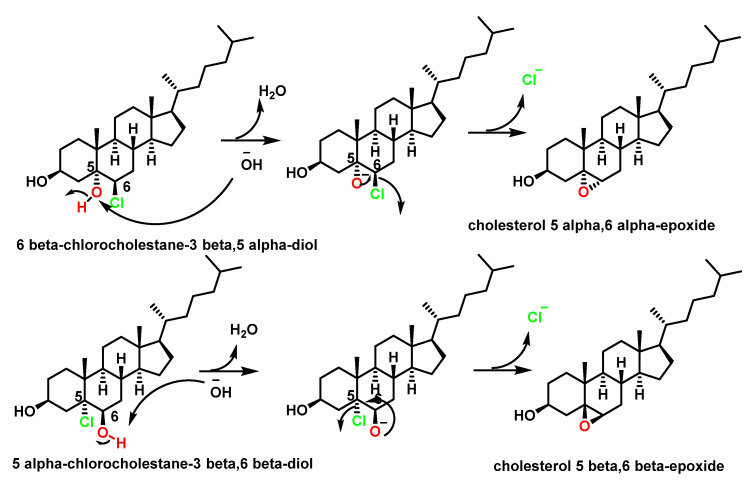
Synthesis of epoxy-cholesterol by intramolecular Williamson reaction via an intramolecular SN_2_ reaction.

The formation of cholesterol chlorohydrin by HOCl has been observed in several cell types including erythrocytes, neutrophils, and mammary carcinoma cells [48]. Unlike aliphatic hydrocarbon chlorohydrins, cholesterol chlorohydrins are unstable and are transformed into epoxides by dehydrochlorination, the mechanism of this reaction being the formation of a chloroalkoxide intermediate by the rapid transfer of a proton to the ^−^OH base, followed by the closure of the cycle with Cl^−^ exit to give the three-linked cyclic ether. Initial deprotonation of the alcohol and the subsequent step are much faster than the attack of the hydroxyl on the chlorine-bonded carbon to the alkanediol, Figure 9.

Chlorohydrins from both unsaturated fatty acids and cholesterol can cause the lysis of target cells, possibly by altering membrane structures. Unlike *N*-chloroderivatives, *C*-chloroderivatives are not oxidising and chlorinating agents.

**Figure 10:**
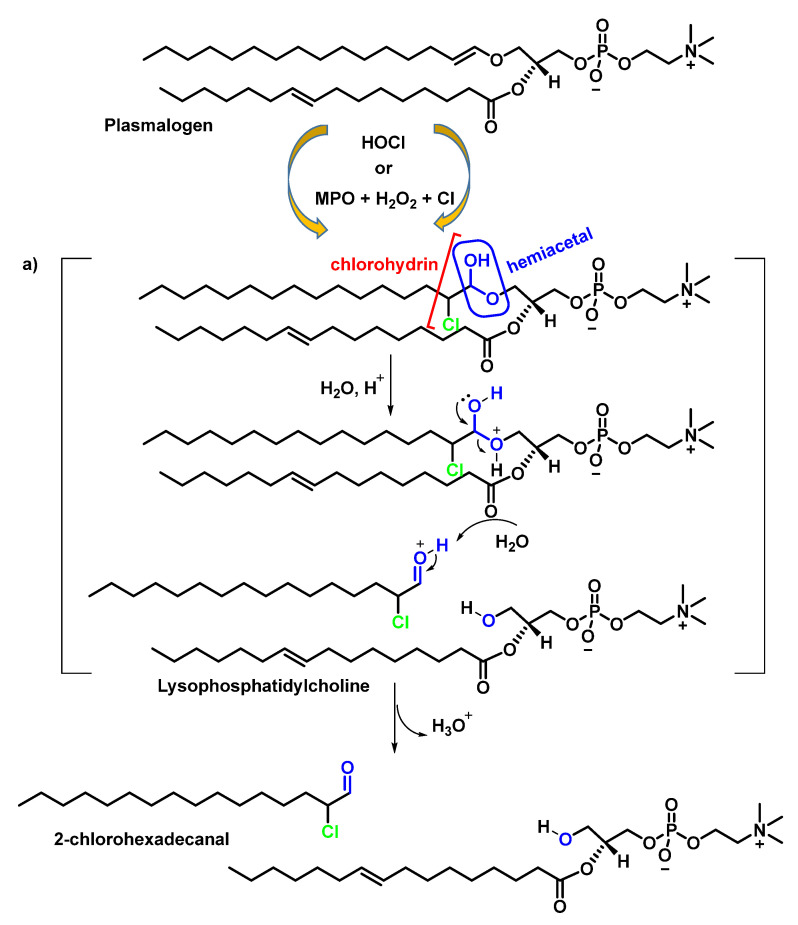
Plasmalogen modification by HOCl or MPO/H_2_O_2_/Cl^−^. (**a**) Acid-catalysed hydrolysis of hemiacetal. The vinyl ether bond of plasmalogens is targeted by HOCl resulting in the release of the masked aldehyde as an α-chloro-fatty aldehyde. The other product of this reaction is an unsaturated molecular species of lysophosphatidylcholine.

Plasmalogens are phospholipids containing an aliphatic chain linked to a vinyl ether attached to the sn-1 position of glycerol and are abundant in tissues of the cardiovascular system [49]. Chemically, this vinyl ether is a masked aldehyde that is acid labile (pH ≤ 2). The HOCl oxidation products of plasmalogen derivatives are α-chloro aldehyde and unsaturated molecular species of lysophosphatidylcholine. The α-chloro aldehyde is the precursor of both α-chloro fatty alcohol and α-chloro fatty acid. Both aldehyde and α-chloro-fatty acid accumulate in activated neutrophils. The production of the alpha-chlorinated aldehyde can be explained by the formation of chlorohydrin, preferentially at the vinyl double bond (C=C–O), which has a higher electron density than C=C. A hemiacetal is obtained which, by acid hydrolysis, fragments and leads to the formation of the aldehyde and the lysophosphatidylcholine alcohol, as shown in Figure 10.

**Figure 11:**
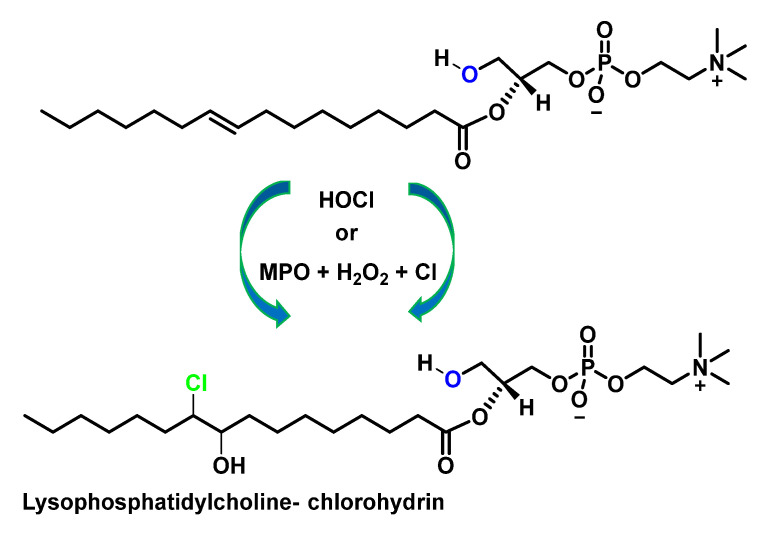
The alkene bond in the unsaturated molecular species of lysophosphatidylcholine is targeted by HOCl to yield a chlorohydrin molecular species.

The reaction continues towards the formation of chlorohydrin from lysophosphatidylcholine, as can be shown in Figure 11.

**Figure 12:**
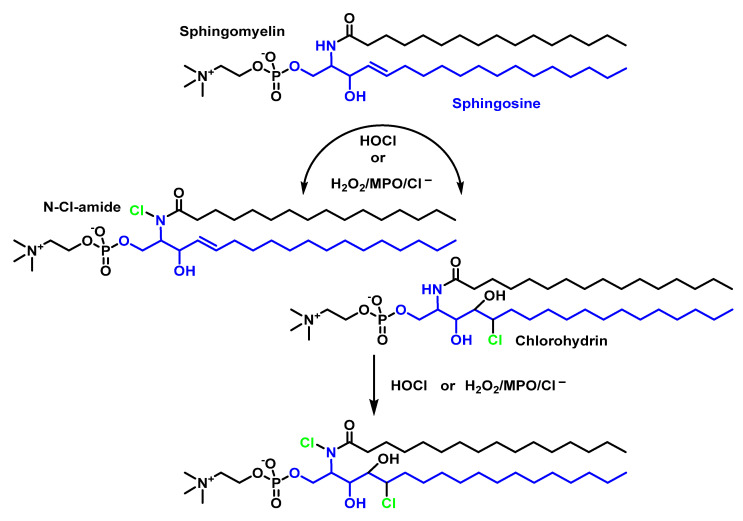
Sphingomyelin modification by HOCl or H_2_O_2_/MPO/Cl^−^. Chlorinated sphingomyelin derivatives produce significant deleterious effects on cells, including apoptosis and DNA damage.

The second most abundant phospholipid in LDL after phosphatidylcholine (~900 µg per mg of protein) is sphingomyelin [50] (~400 µg per mg protein). Sphingomyelin is the only human phospholipid that does not contain glycerol but has the amino alcohol sphingosine. The fatty acid of sphingomyelin is bound to sphingosine by the amino group, while the polar group of phosphocholine is bound to the hydroxyl group of sphingosine. The HOCl targets on the sphingomyelin molecule are the sphingosine double bonds and the hydrogen atom of the amide. The rate constant of the reaction of HOCl with sphingomyelin was estimated to be 18.7 ± 3.05 M^−1^ s^−1^ [51], which is faster than the reaction between HOCl and unsaturated acyl chain bonds (0.56 M^−1^-s^−1^) [52], as shown in Figure 12.

### 4.2. HOCl Reaction with Phospholipids

The polar heads of phospholipids in LDL are mainly choline phosphate, ethanolamine phosphate, and serine phosphate [53]. HOCl does not react with the quaternary nitrogen of phosphatidylcholine [47].

**Figure 13:**
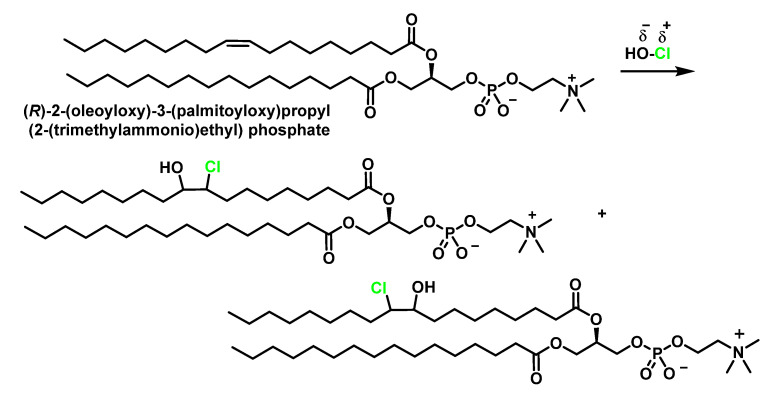
Scheme of reaction of phosphatidylcholine with HOCl.

The unsaturated fatty acids that form phosphatidylcholine, a naturally occurring phospholipid and a major component of the basic structure of biological membranes [54], when chlorinated, lead to the formation of chlorohydrin, which is significantly toxic and can damage cellular structures [40], Figure 13.

**Figure 14:**
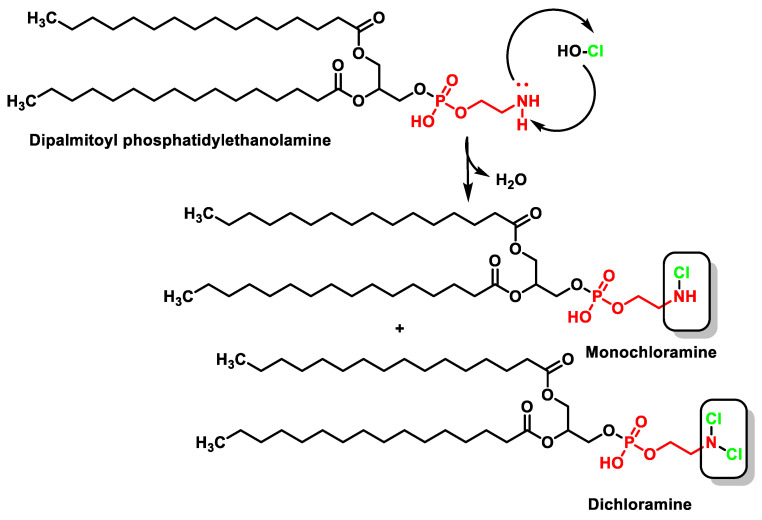
Formation of monochloramine and dichloramine from the oxidation of dipalmitoyl phosphatidylethanolamine by hypochlorous acid.

**Figure 15:**
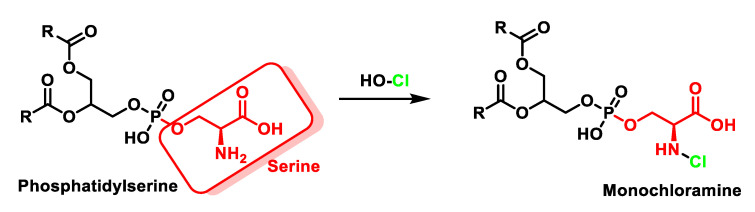
Formation of monochloramine from the oxidation of phosphatidylserine by HOCl.

In phospholipids bearing a primary amino group, such as phosphatidylethanolamine, which is found in all living cells, the formation of chloramines is diminished compared to chlorohydrin, even when there are C=C double bonds in the carbon chains. HOCl transforms phosphatidylethanolamine into mono- and then dichloramine [55], as shown in Figure 14. The oxidation of phosphatidylserine by HOCl is represented in Figure 15.

**Figure 16:**
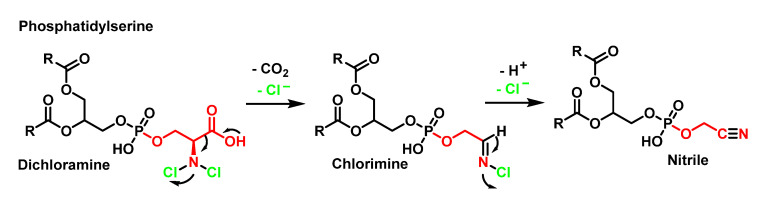
Decomposition of dichloroamine, formation of a transient (chloro)imine.

The decomposition of dichloroamine with the loss of Cl^−^ and decarboxylation gives the formation of a transient (chloro)imine which loses Cl^−^ and a proton to give the nitrile. These modifications by HOCl coincide well with the known pathways for amino acids, as shown in Figure 16.

**Figure 17:**
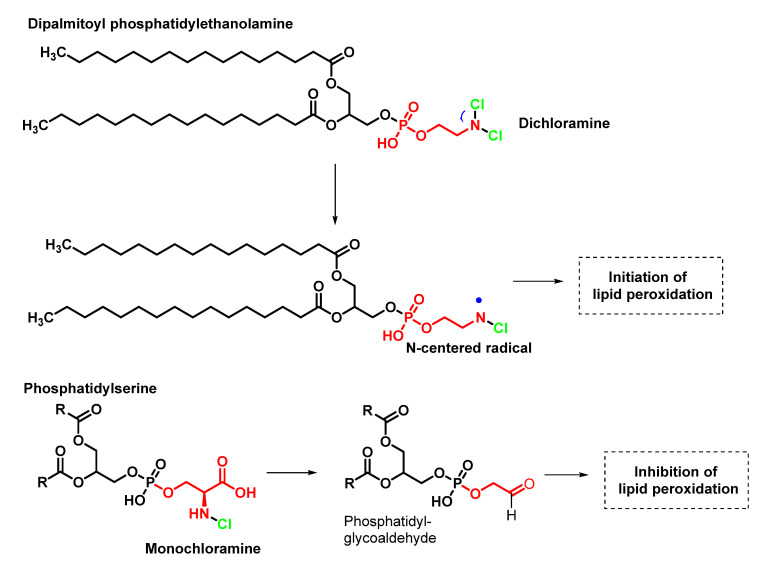
Conversion of the amino group of phosphatidylethanolamine and phosphatidylserine by reaction with HOCl.

Phosphatidylethanolamine and phosphatidylserine chloramines differ considerably in their stability due to the presence of the carboxyl group in the position close to the NH_2_ group on the serine phosphate. The dichloramine of phosphatidylethanolamine undergoes dehydrochlorination accompanied by the homolytic cleavage of the N–Cl bond with the formation of the *N*-centred radical. The chloramine of serine phosphate rapidly decomposes with the formation of aldehydes [55], as shown in Figure 17.

Kawai et al., 2006, indicated that although phosphatidylserine is also chlorinated upon reaction with HOCl, the chloramine formed is rapidly decomposed into phosphatidylglycolaldehyde, a new class of lipid aldehyde. Phosphatidylglycolaldehyde formation is also confirmed in porcine brain PS and in the cell membrane ghost of erythrocytes exposed to HOCl [55]. It is most likely that the rapid formation of the aldehyde, which is stable, prevents the formation of the N radical and thus prevents peroxidation.

## 5. Reaction of HOCl with Ammonia and Aliphatic Amines

The usual behaviour of hypochlorous acid towards carbon and nitrogen in organic substrates is as an electrophilic agent, where chlorine takes on the partial characteristics of the “Cl^+^” ion and combines with the electronic pair of the substrate, simultaneously releasing the OH^−^ ion, often assisted by the H^+^ of the solvent or by other reactive centres of the substrate [56].

One type of HOCl capture compounds of great utility in MPO studies, and which may be the most important under physiological conditions, are nitrogen compounds, particularly ammonia and primary amines. HOCl reacts with these compounds to give the corresponding *N*-chloramines, which also have oxidising and chlorinating activity. The chloramines act as carriers and reservoirs for the “Cl^+^” formed in the MPO-catalysed oxidation of Cl^−^ ions. The most important role of HOCl and chloramines in leukocytes is to mediate the toxicity of the MPO/H_2_O_2_/Cl^−^ system on the target cells of these oxidising agents, usually micro-organisms that have invaded the tissues [57].

**Figure 18:**
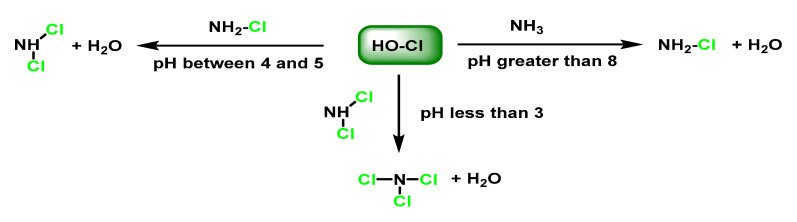
Synthesis of mono, di, and trichloro ammonia.

The chlorination of ammonia gives monochloramine NH_2_Cl and aliphatic amines give the derived monochloramines RNHCl. *N*-chloramines are reactive and have a long half-life and are strong oxidisers and chlorinating agents, although they are less reactive than HOCl and are not reduced by H_2_O_2_ [58]. Ammonia and amines protect MPO from inactivation, indicating that chloramines do not attack the enzyme. With ammonia, for example, NH_2_Cl, NHCl_2_, and NCl_3_ are sequentially formed via the reactions, as shown in Figure 18.

The formation of the different species is conditioned by the ratio of chlorine/ammonia concentrations, when the concentration of ammonia and amines is higher than the amount of HOCl, the formation of chloramines with a single N–Cl bond is favoured. The reaction time, the temperature as well as the pH of the reaction medium also play a role. Thus, at pH values below 3, mainly tri-chloramine is obtained, and at pH values between 4 and 5, dichloramine is formed and the product that is mainly generated at pH > 8 is monochloramine [59,60].

**Figure 19:**
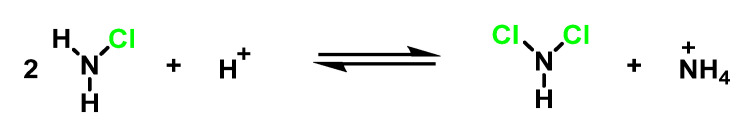
Dihaloamines obtained by the disproportionation reaction of the corresponding *N*-monohaloamine in an acid medium.

Dihaloamines can also be obtained by the disproportionation reaction of the corresponding *N*-monohaloamine in an acid medium, through the reaction of Figure 19.

For reactions of HOCl with the N atom of amines and similar compounds, the electrophilic chlorine atom could be visualised as trapping the free electron pair of the N atom releasing the H^+^ of the amine and the OH^−^ of HOCl. Alternatively, the reaction could be interpreted as a nucleophilic displacement of the OH^−^ from the HOCl by the amine in which case the nucleophilicity of the amine will compete with that of the OH^−^ ion. According to either reaction model, the specific chlorination rates on amines vary directly with the basicity (or nucleophilic character) of the nitrogenous substrate.

In the case of primary aliphatic amines, mono- and di-chloramines RNHCl and RNCl_2_, respectively) are formed, while in the case of secondary aliphatic amines, only monochloramine R_1_R_2_NCl formation is possible. The reaction rate is also fast and increases with the nucleophilic character of the amine attacking the chlorine atom of the HOCl. The chlorination of tertiary amines is at least three orders of magnitude slower than the chlorination of secondary and primary amines of similar basicity. This behaviour is explained based on the unavailability of hydrogens of these amines.

**Figure 20:**
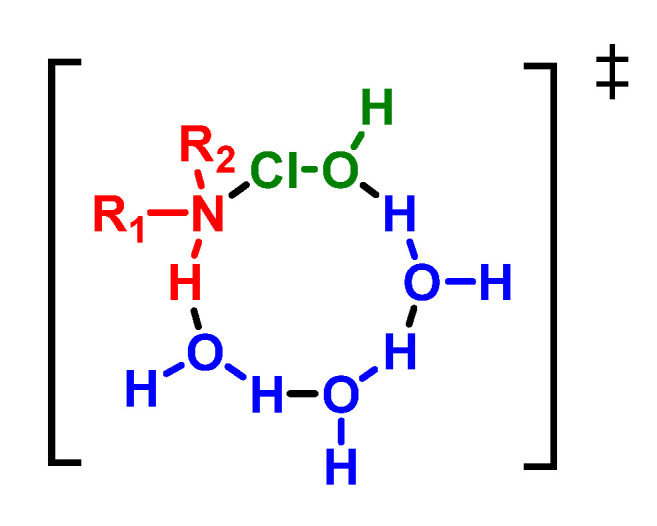
Transition state of the amine chlorination reaction.

A recent theoretical study on the chlorination of *N*-compounds indicates that the transition state involves three water molecules to give rise to a concerted cyclic transition state [61,62,63], Figure 20.

Chloramines represent an important class of leukocyte oxidants and contribute to microbicidal, cytotoxic, and cytolytic oxidative activities, the chemical modification of regulatory substances, and the uptake and metabolism of nitrogenous compounds [64]. The most abundant *N*-residues available for reaction with HOCl in biological systems are primary amino groups, such as taurine, polyamines, amino sugars, lysine, and extreme residues of proteins.

## 6. Reactions of HOCl with Amino Acids

Hypochlorous acid is a weak acid and its pKa is 7.6 [65]; however, it is an extremely strong oxidant that reacts indiscriminately with a wide variety of functional groups in proteins, nucleotides, and lipid membrane, and selectively oxidises thiols [66]. In the case of proteins, the oxidising HOCl most rapidly reacts with sulphur containing residues such as cysteine and methionine, followed by protein side chains containing lysine, histidine, tryptophan, and tyrosine [67].

### 6.1. Reaction of HOCl with Nitrogen-Containing Compounds

HOCl rapidly reacts with nitrogen-containing compounds (particularly amines and, to a lesser extent, amides), producing chlorinated derivatives, including chloramines (R–*N*(R′)–Cl) and chloramides (R–C(O)–*N*(R′)–Cl). These compounds can be further oxidised to form dichlorinated species (R–*N*–Cl_2_).

In addition, nitrogen chlorination can occur at the α-amino group of amino acids, at the *N*-terminus of peptides and proteins, and at nucleophilic centres on the side of protein chains (e.g., lysine, arginine). On the other hand, chloramides can be generated at the peptide bonds of proteins and, in excess of HOCl, at the side chains of asparagine and glutamine, with amide groups. The formation of chloramines interferes with protein folding and leads to protein aggregation.

**Figure 21:**
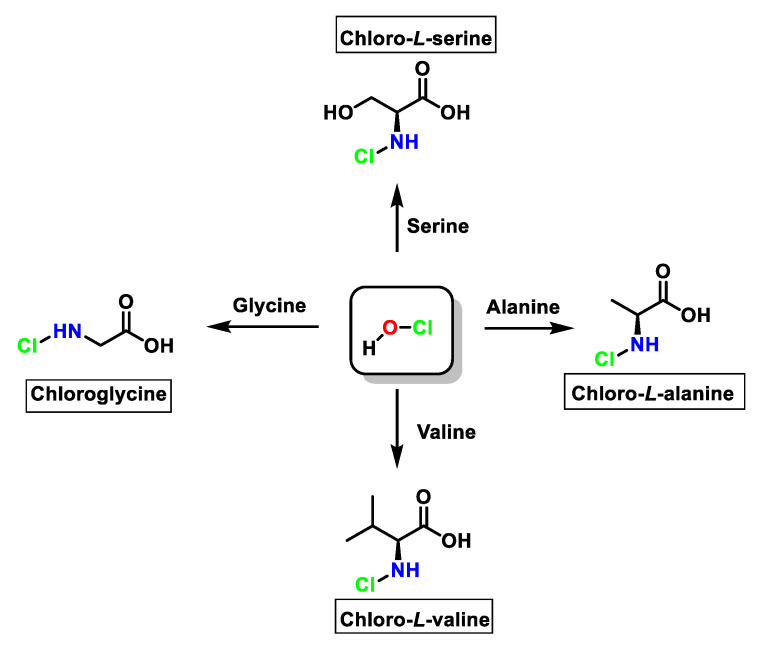
Schematic the mechanism of formation of *N*-chloro-α-amino acids from amino acids includes an electrophilic attack via the Cl^+^ of HOCl on the nitrogen atom together with a proton transfer from the nitrogen atom to HOCl to release a water molecule.

For amino acids without a functional group in the chain that can react with HOCl (Gly, Ala, Val, Ser), the reaction proceeds only with the amino group with monochloramine formation, and dichloramine is formed in excess of HOCl, as shown in Figure 21.

### 6.2. Reaction of HOCl with Aromatic Amino Acids

**Figure 22:**
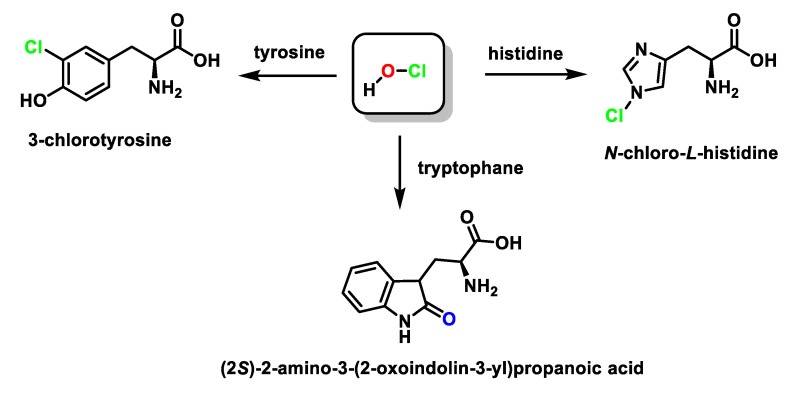
Products obtained in the reaction of aromatic amino acids with HOCl.

The imidazole ring of histidine reacts with HOCl to form a short-lived chloro-amine, which rapidly transfers its chlorine group into another amine [68]. Tryptophan reacts with HOCl to form 2-oxindole, while the reaction of HOCl with tyrosine forms 3-chlorotyrosine and 3,5-dichlorotyrosine, Figure 22.

**Figure 23:**
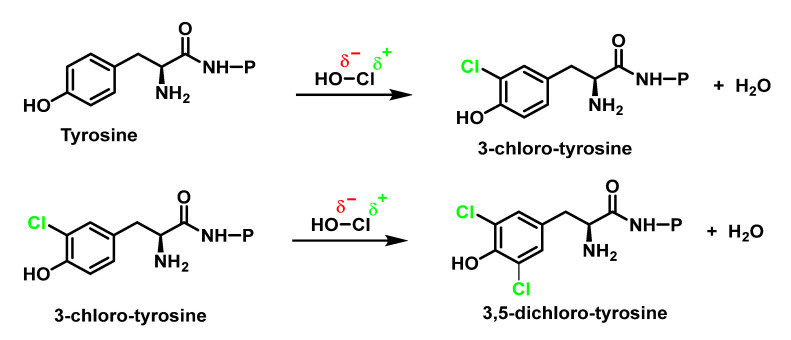
Reaction of P-protected tyrosine with HOCl and formation of 3-chloro- and 3,5-dichlorotyrosine derivatives.

3-Chlorotyrosine is a chemically stable product formed when tyrosine reacts with HOCl, or a chloramine generated by HOCl. In particular, it has been observed that this aromatic amino acid (both free and in protein) undergoes the rapid chlorination of the aromatic ring, through an electrophilic aromatic substitution reaction of a hydrogen for a chlorine to give 3-chlorotyrosine with excess HOCl to form 3,5-dichlorotyrosine [69,70,71,72,73,74], as shown in Figure 23.

Direct HOCl attack and a chlorine transfer from the chloramine initially produced in the amino group of the amino acid are the two mechanisms proposed for the chlorination of the phenolic side chain of Tyr [75].

**Figure 24:**
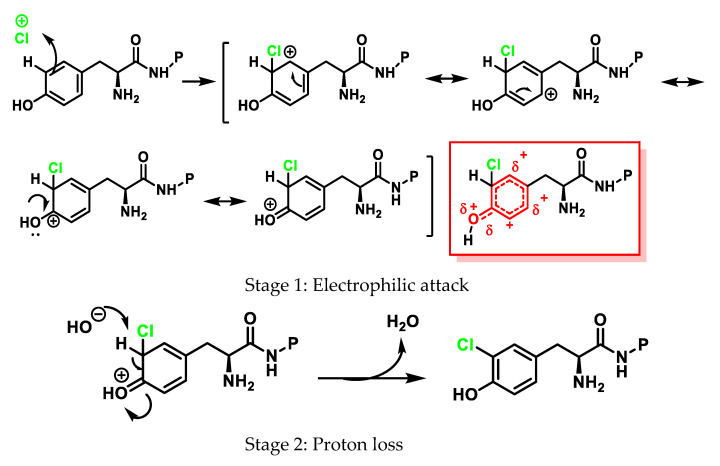
Mechanism of the electrophilic substitution in tyrosine and the formation of 3-chlorotyrosine. Stage 1: electrophilic attack; Stage 2: proton loss.

The general mechanism of electrophilic aromatic substitution begins with the electrophilic attack of “Cl^+^” to give an intermediate hexadienyl cation with delocalised charge, in a step that is the rate-determining Stage 1, as shown in Figure 20. The subsequent rapid loss of protons regenerates the aromatic ring (now substituted), as shown in Stage 2 of Figure 24.

Hypochlorous acid and chloramines result in the chlorination of tyrosine residues to form 3-chlorotyrosine [76]. This halogenated amino acid is a specific biomarker for HOCl-mediated damage [39].

**Figure 25:**
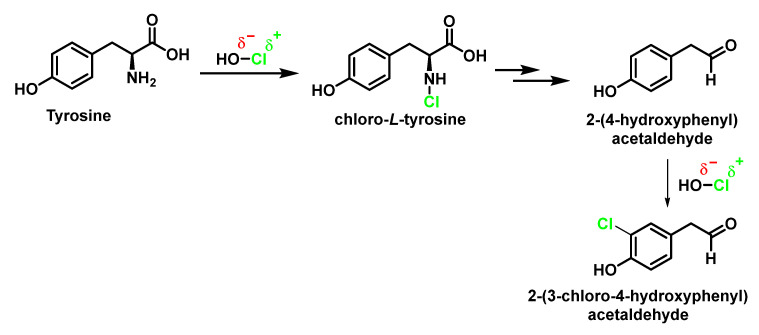
Formation of p-hydroxyphenylacetaldehyde [77] and 3-chloro-4-hydroxyphenylacetaldehyde.

With the free amino acid, in addition to the reaction with the aromatic ring to give 3-chlorotyrosine, a reaction can occur at the amino group and the resulting formation of p-hydroxyphenylacetaldehyde [77]. Both products can continue to react to give, respectively, 3-chloro-4-hydroxyphenylacetaldehyde and 3,5-dichlorotyrosine [73], Figure 25.

**Figure 26:**
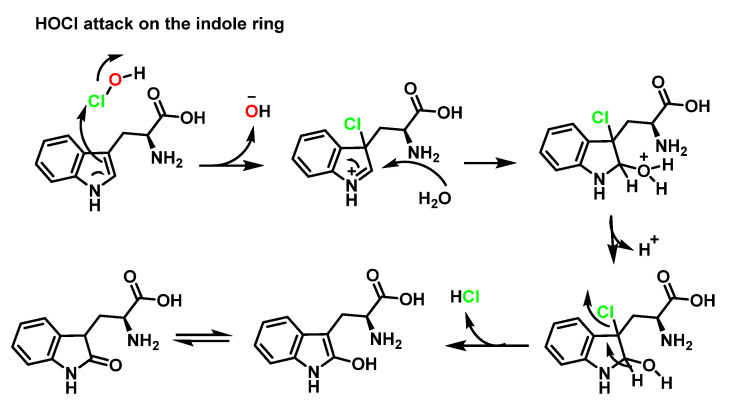
Hypochlorous acid could directly attack the free amino group of the amino acid Trip, with the consequent formation of a chloramine, in an electrophilic substitution mechanism [78].

HOCl-mediated oxidation of free tryptophan can proceed via direct attack by HOCI on the indole ring, for example, in a classical electrophilic aromatic substitution mechanism such as that of tyrosine. Chlorine enters the C–3 position of the indole ring forming an iminium cation, which is attacked at C–2 by a water molecule, after the removal of HCl, aromaticity is restored and the enol is obtained, which is in equilibrium with the carbonyl, as shown in Figure 26.

**Figure 27:**
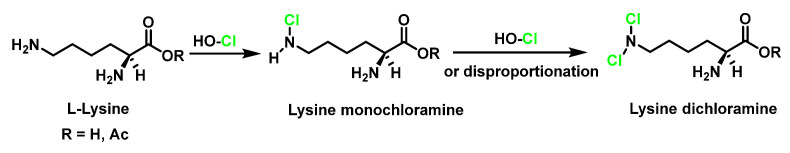
HOCl reaction with Lys and formation of monochloroamine and dichloroamine.

Lysine and arginine are the second targets of HOCl in proteins, which chlorinates them to form chloramines. Lys and Arg are chlorinated by HOCl or MPO/H_2_O_2_/Cl^−^ at the N of the amino acid chain, as shown in Figure 27.

**Figure 28:**
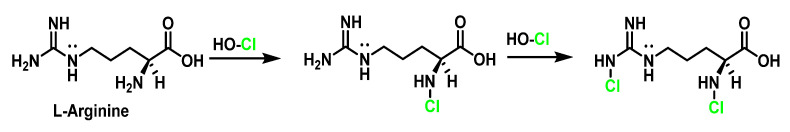
Proposed structures of chlorinate L-arginine products.

Using spectrophotometric techniques, HOCl and L-Arg were found to react rapidly (k = 7.1 × 10^5^ m^−1^ s^−1^) to form two main products which were identified by mass spectrometry as monochlorinated and dichlorinated adducts of L-Arg [79], as shown in Figure 28.

### 6.3. Reaction of HOCl with SULFUR-Containing Amino Acids

**Figure 29:**
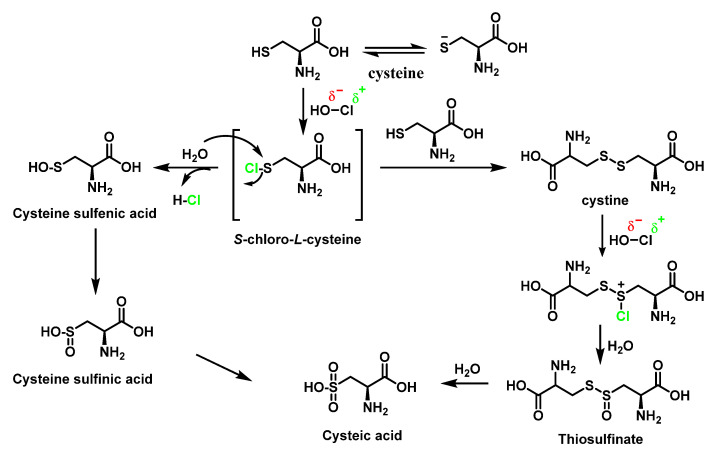
Summary of products of oxidation of Cys and Cystine residues by HOCl.

HOCl and reacts extremely rapidly (k ≈ 3107 M^−1^ s^−1^) with sulphur-containing residues (cysteine and methionine) [75,80]. The thiol functional group of the amino acid cysteine can undergo a wide range of oxidative modifications. The thiol group of cysteine reacts with HOCl and through the formation of an unstable sulphenyl chloride which undergoes various reactions with nucleophilic reagents can react with water to form a sulphenic acid Cys-SOH [73,78,81]. Most sulphenic acids are highly unstable, with a half-life of minutes and that react with a cysteine thiol present in the vicinity to form a disulphide or are further oxidised to sulphinic Cys-SO_2_H and sulphenic Cys-SO_3_H acids [82]. Sulphenyl chloride can react with the thiol group of a second cysteine molecule to generate the disulphide, cystine. Cystine can also be oxidised by HOCl, possibly via an intermediate sulfenyl chloride CysS^+^(Cl)SCys, to finally give rise to more cysteic acid by hydrolysis, as shown in Figure 29.

**Figure 30:**
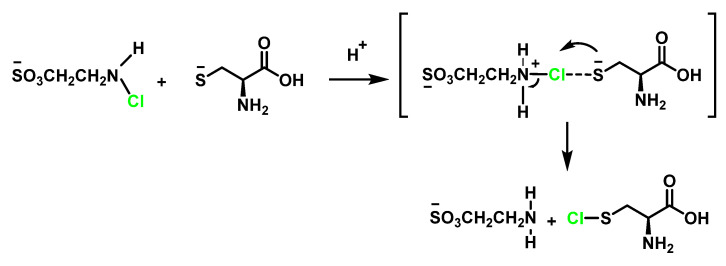
Formation of S-chloro-L-cysteine.

Cysteine can also react with *N*-Cl-taurine to generate sulphenyl chloride, as shown in Figure 30.

**Figure 31:**
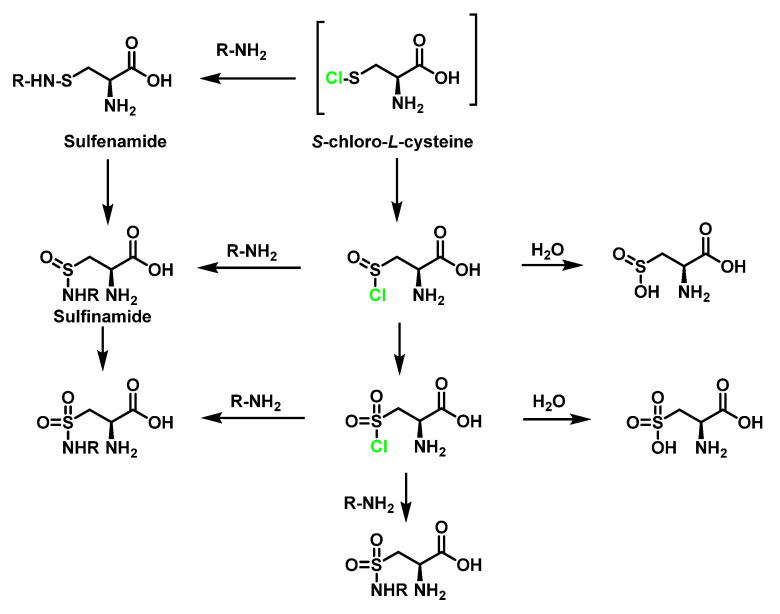
Proposed reaction pathways for S–*N* cross-linking and oxidation by HOCl of Cys.

Sulphenyl chloride intermediates can react with nitrogen derivatives [83]. The guanidine groups of the Arg side chains, and the side-chain amines of Lys are all capable of reacting with sulphenyl chlorides to form sulphenamides RSN-. Other reactions of sulphenamides with oxygen produce sulphinamides Cys-SO-N-R and sulphonamides Cys-SO_2_-*N*-R. Chloride Cys-S-Cl is converted into sulphonyl chloride Cys-SO_2_-Cl, in excess of HOCl. Sulphonyl chloride Cys-SO_2_Cl can be converted to thiosulphonates Cys-SO_2_-S-Cys on reaction with thiols Cys-SH. Both sulfonyl chloride Cys-SO_2_Cl and thiosulfonates Cys-SO_2_-S-Cys can be hydrolysed to form sulfonic acid Cys-SO_3_H. Generally, the irreversible formation of sulphonic acid Cys-SO_3_H inhibits protein formation or degrades proteins. In addition to the aforementioned reactions, sulfonyl Cys-SO_2_Cl and sulfenyl Cys-S-Cl chlorides can also react with amino R-NH_2_ compounds to form sulphenamide Cys-S-NH-R, sulphinamide Cys-SO-NH-R, as well as sulphonamide Cys-SO_2_-NH-R. In addition, sulphenyl chlorides Cys-S-Cl can decompose to thiyl radicals Cys-S at a high temperature, in the presence of metal ions or ultraviolet light [84]. This process can compete with the other processes described above, particularly when the thiol group is isolated or within a protein, as shown in Figure 31.

**Figure 32:**
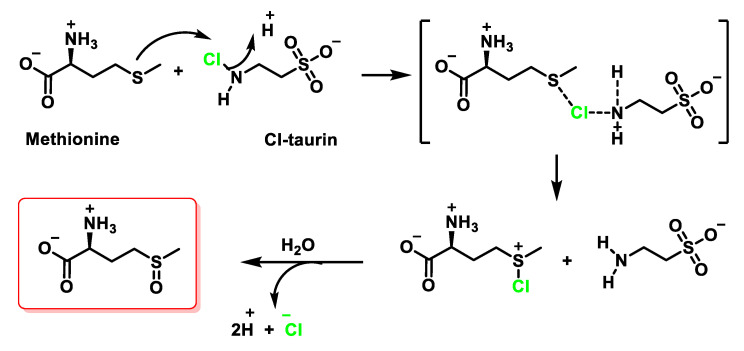
Mechanism of Met oxidation mediated by Cl–taurine to yield methionine sulfoxide.

The oxidation of the Met side chain by HOCl is less complex, where methionine sulfoxide is the only product observed with even a 2.5-fold molar excess of HOCl over *N*-acetyl-Met [85]. Methionine sulfoxide is a stable oxidation product of Met, and it is likely that this oxidation plays a critical role in the bactericidal action of HOCl. The reaction of methionine with HOCl initially produces a sulphonium chloride, which is subsequently hydrolysed to give sulfoxide as the final product. Assuming that the same product is formed with chloramines, then a mechanism can be proposed in which hydrogen ions promote the transfer of Cl^+^ to sulphur [86], as shown in Figure 32.

Proteins, the most abundant cellular molecules, are the main target of HOCl. In this context, sulphur-containing compounds such as cysteine, methionine and glutathione have the highest degree of reactivity with HOCl compared to other biological molecules. Thiols are cell crucial targets for oxidation.

Second-order rate constants were determined by Pattison and Davies, 2001, for the reactions of HOCl with amino acids side chain groups, α-amino groups and backbone amides, Table 1, at physiological pH, 7.4, in aqueous solution. The order of reactivity of the various side chains was Met > Cys > Cystine > His > Trp > Lys > Tyr > Arg > Gln > Asn [80]. Constants are second-order determined for the reactions of HOCl with amino acids’ side-chain groups, α-amino groups, and backbone amides [79].

### 6.4. Reaction of HOCl with Taurine

Taurine, 2-aminoethane sulphonic acid, was first isolated in 1827 by Friedrich Tiedemann and Leopold Gmelin [87] in ox (bull) bile, hence its name. Taurine, 2-aminoethanesulphonic acid, is a β-amino acid containing sulphur which is not involved in protein synthesis, but is abundant in the cytoplasm of most cells [88]. Taurine is involved in the elimination of foreign micro-organisms by the immune system [89]. Its other functions include its role as a neurotransmitter, protector of the photoreceptors of the retina, antioxidant, etc.

Taurine is an amino acid found in high concentration in cells, acting as a trap for HOCI generated by the MPO/H_2_O_2_/Cl^−^ pathway to form *N*-Cl-taurine, which can still be an oxidant with much lower reactivity and toxicity than HOCl. Taurine chloramine possesses antimicrobial and anti-inflammatory activity [90]. Taurine is present in human plasma and cells, acts as a major HOCl-attractant forming taurine chloramine (–O_3_SCH_2_CH_2_NHCl), is more stable and a weaker oxidant. These species exert a protective effect on neutrophils and help retain bactericidal potential for prolonged periods. Recent studies have suggested that chloro-taurine may act as a physiological modulator of inflammation, inhibiting the production of pro-inflammatory agents [91].

**Figure 33:**
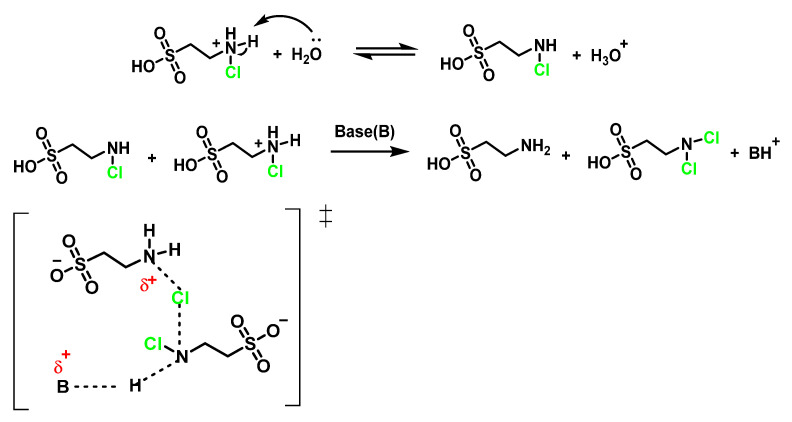
Proposed transition state for the *N*-chlorotaurin disproportionation reaction.

Tau-NHCl, which accumulates in the extracellular medium and does not inhibit neutrophil functions, may continue to moderate neutrophil cytotoxic activity long after HOCI has been eliminated. Antelo et al., 2000, have studied the disproportionation of *N*-chlorotaurin Tau-NHCl to give rise to N, *N*-dichlorotaurin Tau-NCl_2_. These authors suggest that the abstraction of a proton for a base takes place in the slow stage and propose a concerted mechanism in which deprotonation and chlorine transfer occur simultaneously in the transition state [92], as shown in Figure 33.

Chlorinated taurine derivatives are more hydrophilic and do not pass through biological membranes, so they have very low toxicity [93]. Taurine-chloramine TauCl also appears to play a chlorinated oxidant scavenger role. Taurine chloramines react slowly with bactericidal components, and taurine obstructs the bactericidal activity of the MPO/H_2_O_2_/Cl^−^ pathway [94]. Leukocytes contain 20–30 mM taurine in the cytosol [95], and it is proposed that leukocytes use taurine to trap HOCl from phagolysosomes [90]. Taurine derivatives, *N*-chlorotaurine TauNHCl, and N, *N*-dichlorotaurine TauNCl_2_, are the most important components formed by the reaction of chlorine generated by the MPO/H_2_O_2_/Cl^−^ pathway with taurine, an amino acid found in the body in higher concentrations than other amino acids.

### 6.5. Reaction of HOCl with Peptides and Proteins

The above reactions of HOCl with functional groups on amino acid chains can also occur in the case of proteins and polypeptides. Proteins are likely to be the main targets for HOCl reaction within a cell due to their abundance and high reactivity with HOCl, although little information is available on the oxidation mechanisms [80].

Within proteins, the sulphur of cysteine Cys and methionine Met react rapidly with HOCl, but also the side chains of lysine (Lys), histidine (His), tryptophan (Trp), and tyrosine (Tyr), and the -amino groups of amino acids and peptides are oxidised and/or chlorinated. However, the reactivity of functional groups in amino acid side chains and the same groups in the structure of a polypeptide chain can vary significantly. The treatment of small globular proteins, such as insulin (5.7 kDa) and lysozyme (14.4 kDa), with increasing concentrations of HOCl, leads to modification of several amino acid residues (His, Lys, Arg, Tyr, etc.). The tertiary structure of the protein influences the reactivity of the amino acid residue side chains and/or their availability for HOCl.

**Figure 34:**
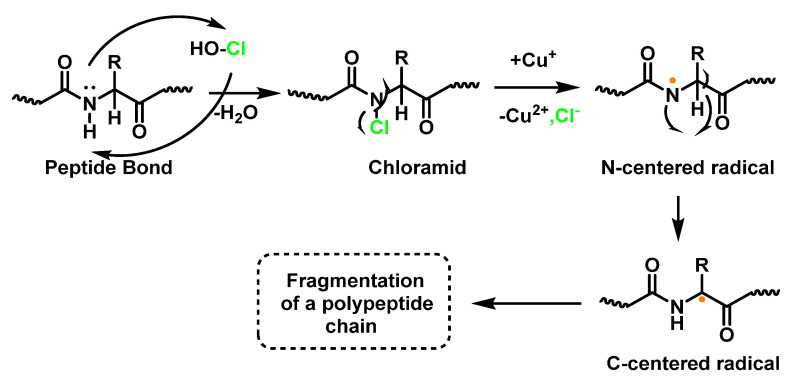
Formation of chloramide.

The result of the HOCl-protein interaction is fragmentation, which is mainly due to the cleavage of a peptide bond. When HOCl interacts with a peptide bond, the chloramide is formed, as can be seen in the scheme. It should be noted that the reaction rate with the amide of the peptide bond is much slower than with an amino group. The reaction rate constant depends to a large extent on the chemical structure of the compound. In aqueous media, chloramines hydrolysis slowly with peptide bond breakage and protein fragmentation [96]. In addition, the *N*–Cl bond can undergo homolytic cleavage with the formation of an *N*-centred radical, e.g., in the presence of transition metal ions. The *N*-centred radical is short-lived, these species undergo rapid migration reactions of 1,2 hydrogen atoms to give α-carbon centred radicals stabilised by C=O conjugation with most peptides, leading to the fragmentation of the polypeptide chain [97], as shown in Figure 34.

**Figure 35:**
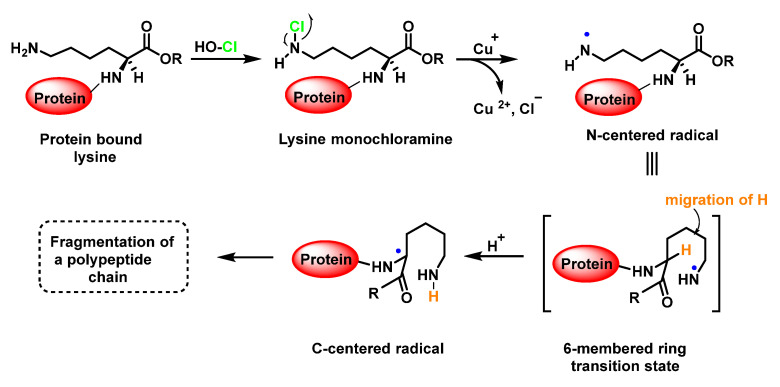
Formation of the radical centred on the N of Lys with subsequent transformation to a C-centred radical.

Another reason for the cleavage of the peptide bond may be the formation of chloramide or chloramine in the reaction of HOCl with the side-chain amide group of Glutamine or with the amino group of Lysine [98], as shown in Figure 31, in the case of Lysine, with intramolecular reordination via a six-link cyclic intermediate, transformed into a radical centred on the C atom of the peptide bond. Under aerobic conditions, such a C-centred radical is rapidly converted into a peroxyl radical with subsequent degradation of the polypeptide chain, Figure 35.

### 6.6. Reaction of HOCl with Glutathione

Glutathione GSH is a water-soluble, low-molecular-weight tripeptide that is present in millimolar concentrations in almost all eukaryotic cells and in many bacteria, most of which are Gram-negative. Glutathione is synthesised by the consecutive action of two ATP-dependent enzymes.

While glutathione serves as a redox buffer, the oxidation of the sulphydryl groups on proteins can affect their functional properties. The formation of protein di-sulphides, mixed disulphides with glutathione or sulphenic acids can lead to changes in enzymatic activity, conformation or affinity towards other molecules. Such changes contribute to cell damage caused by oxidative stress. The modulation of the thiol redox state also provides a sensitive mechanism for the regulation of metabolic processes of the thiolate group [99], RS-, which is more reactive as a nucleophile than the corresponding protonated group RSH.

Glutathione GSH is a classic example of a scavenger antioxidant. It forms the first line of defence and efficiently removes reactive species, e.g., hypochlorous acid, before they cause damage to biomolecules.

**Figure 36:**
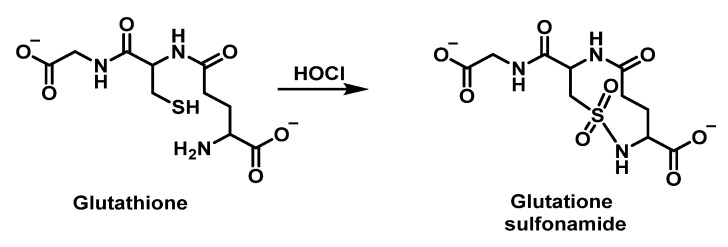
Reaction of GSH with HOCl and formation of glutathione sulphonamide.

Winterbourn et al., 2002, studied the chlorination of GSH by HOCl and found glutathione thiosulphonate GSO2SG and the corresponding sulphonamide as oxidation products alongside GSSG. In this case, intramolecular condensation is thought to occur between a sulphonyl chloride intermediate RSO_2_Cl of Cys and the amino group of the glutamyl side chain. It has been proposed that glutathione sulphonamide may be a specific marker of HOCl oxidation in biological systems. In addition to understanding the impact of reactive oxidants on cellular function, it is important to have reliable biomarkers to detect oxidant formation and its consequences in vivo. Glutathione sulphonamide is a promising candidate for a biomarker of HOCl production by neutrophils in vivo. The reaction with GSH is one of the fastest reactions observed for HOCl, which makes GSH likely to be an important target for HOCl in vivo, as shown in Figure 36.

**Figure 37:**
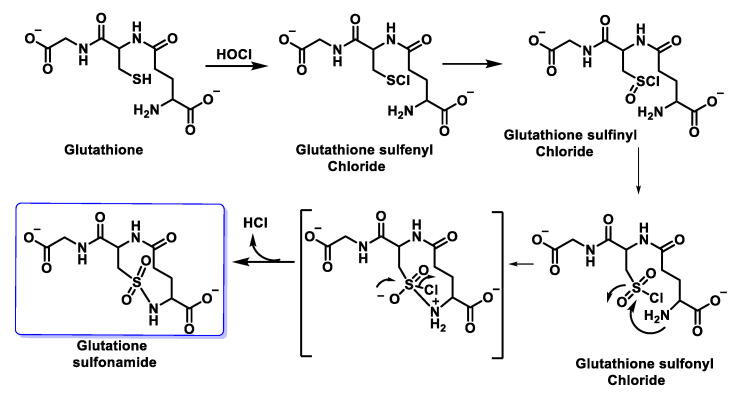
Intramolecular cyclisation of the RSCl intermediate with the terminal amino group on oxidation by hypochlorous acid. The proposed mechanism of formation is through the condensation of the sulphonyl chloride with the amino group of the glutamyl residue of glutathione.

The possible mechanism for this transformation is shown in Figure 37.

In human red cells, GSH is oxidised to glutathione disulphide GSSG; therefore, GSH probably acts as a protector of cells against HOCl toxicity [100]. Vissers et al., 1995, exposed human red blood cells to low doses of hypochlorous acid, resulting in the loss of intracellular GSH. The disappearance of GSH precedes the oxidation of membrane thiols and the formation of chloramines. The lost GSH is converted into GSSG. These authors indicate that in metabolically active cells, GSH is recovered, so it may act as a protector of cell components against HOCl damage [101].

Other more complex oxidation products involving the intramolecular reaction of the RSCl intermediate with adjacent primary or secondary amines to form sulphonamide bonds have also been detected [102].

Sulphenyl chloride GSCI reacts preferentially with more GSH to give rise to GSSG [103]. In a later work, Pullar et al., 2000, working with endothelial cells, obtained GSCI as a major product of GSH oxidation by HOCl. The corresponding cyclic sulphonamide, observing a very small increase in GSSG and glutathione sulphonic acid, both in the extra- and intracellular medium [104].

**Figure 38:**
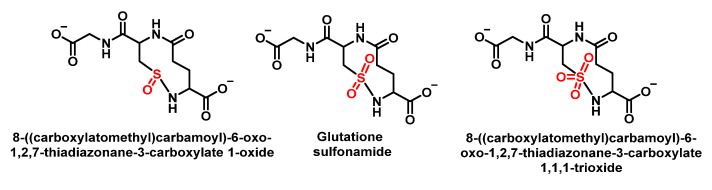
Cyclic amides formed in the reaction of GSH with HOCl.

Following Winterbourn and Brennan, 1997, with the proposal of sulphonamide formation, Haenen et al., 2014, proposed that glutathione has a total scavenging capacity of four HOCl molecules. It appears that one molecule of GSH is rapidly consumed by the reaction with one molecule of HOCl, but the total HOCl scavenging capacity of GSH amounts to four, i.e., four molecules of HOCl are removed in total by one molecule of GSH. GSH is not only a good biological antioxidant because of its reaction rate with HOCl, but because the products generated are also effective antioxidants, as shown in Figure 38.

### 6.7. Degradation of Amines, N-Monochloro-α-Amino Acids, and Lys

Once formed, *N*-halo-derivatives can undergo different processes depending on the conditions of the medium: disproportionation, fragmentation, and elimination. Mono- and dichloramines are unstable and are mild oxidisers that can transfer chlorine to other substrates, regenerating the original amine in the process [102,105]. Reactions of amines, such as Lys side chain or amino terminal groups, with HOCl lead to the formation of unstable mono- and dichloramines (with high excesses of HOCl). These products are mild oxidants and can transfer chlorine to other substrates, regenerating the original amine in the process [106].

**Figure 39:**
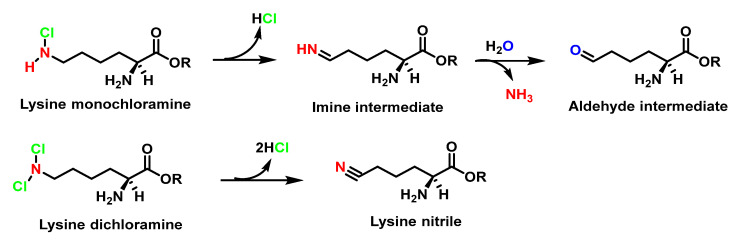
Decomposition of lysine monochloramide with formation of aldehyde and nitrile from dichlorinated Lys.

It has also been proposed that Lys-derived chloramines are transformed into aldehydes by the loss of hydrochloric acid and ammonia, as shown in Figure 39.

**Figure 40:**
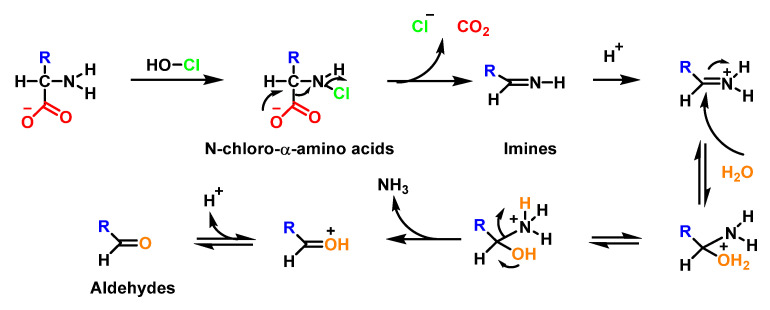
Degradation mechanisms of N-chloro-α-amino acid anions under neutral conditions.

In the absence of oxidisable substrates, the chloramines formed in amino-acids decompose to produce aldehydes and amines under neutral conditions [73]. This is believed to occur through decarboxylation and the loss of chloride from chloramines to form unstable imines which undergo acid hydrolysis to generate aldehydes with the loss of ammonia [107], as shown in Figure 40.

**Figure 41:**
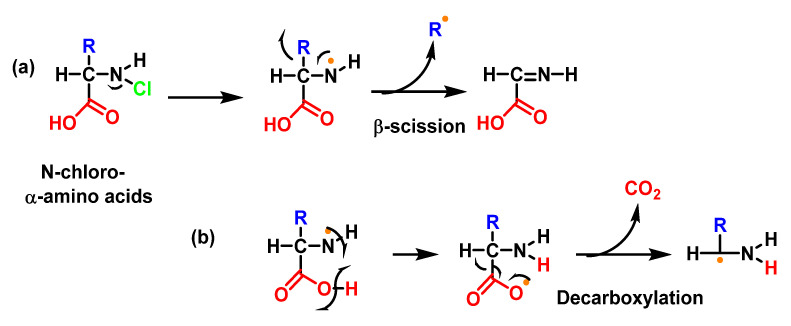
(**a**) β-fragmentation reaction by homolytic cleavage of the C-C bond in position to an N-centred radical, giving rise to an iminic derivative and a C-radical R; and (**b**) β-fragmentation by homolytic cleavage with the loss of CO_2_ and a C-centred radical.

Studies with Lys and other amino acids have revealed that the initial nitrogen-centred radicals can undergo a number of additional rapid rearrangements, e.g., for example, cleavage processes with a loss of the side chain or carboxyl groups, as shown in Figure 41.

**Figure 42:**
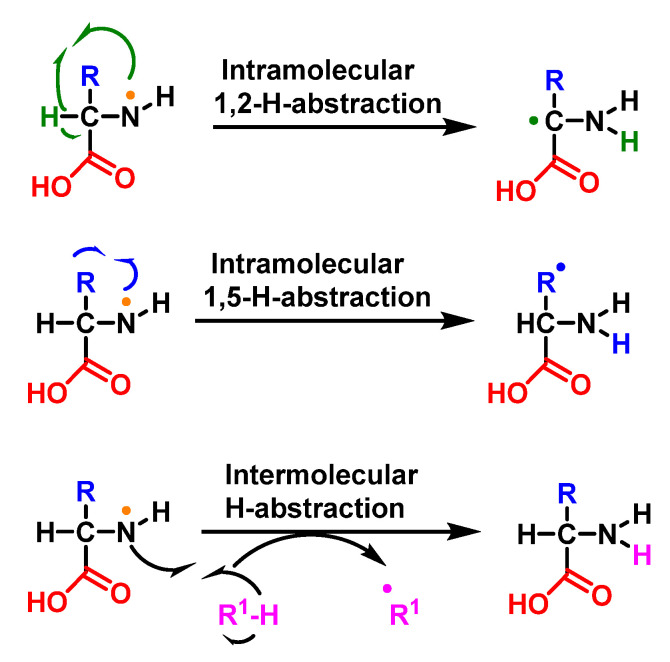
Intra- or intermolecular hydrogen abstraction reactions.

Another reaction is that of the intra- or intermolecular hydrogen abstraction, a 1,2, 1,5 (and sometimes 1,6) transfer of a hydrogen atom from a delta or epsilon carbon to the amine radical or other species capable of readily transferring an H, giving rise to a C-radical, as shown in Figure 42.

## 7. Reactions of HOCl with Nucleotides and Nucleic Acids

**Figure 43:**
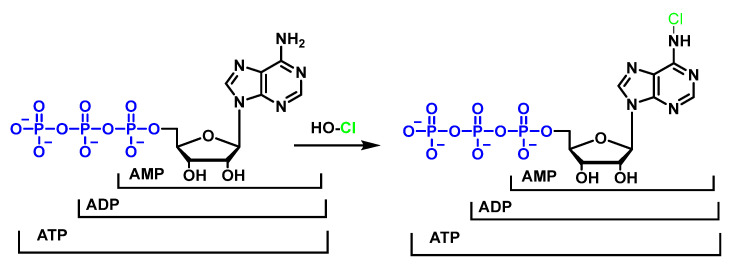
Reaction of AMP/ADP/ATP with HOCl and chlorination at the primary amino group of the adenosine derivative.

Studies on the reactions of HOCl with nucleotides and nucleic acid bases indicate that the most favoured reactions are with amino groups to give chloramines [108,109]. Investigations of the reactions between HOCl and nucleotides have led to the proposal that HOCl exerts its toxic action by destroying the electron transport chain and the adenosine nucleotide pool AMP/ADP/ATP [110,111], as shown in Figure 43.

**Figure 44:**
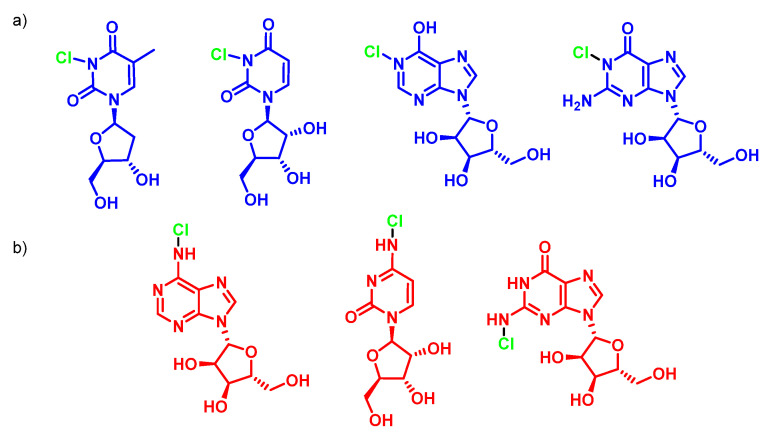
(**a**) Faster NH-chlorination of thymidine, uridine, inosine, and guanosine; and (**b**) slower –NH_2_ chlorination of adenosine, cytidine, and guanosine.

HOCl does not react with ribose and therefore the most likely target for HOCl on nucleotides is the nitrogen in the nitrogenous bases. It has been shown that the HOCl-induced chlorination of the –NH_2_ substituents of nucleotides available on adenosine, cytidine and guanosine is much slower, in neutral solution, than the chlorination of the heterocyclic –NH groups available on guanosine, inosine, thymidine, and uridine. These secondary chloramines, –NCl, are particularly reactive, for example, with GSH, disulphide, NADH, and aliphatic amines, as shown in Figure 44.

Experiments with nucleoside mixtures show that the propensity for radical formation is cytidine > adenosine = guanosine > uridine = thymidine heterocyclic chloramines are themselves reactive and capable of acting as chlorine donors. Therefore, these products are not suitable biomarkers. However, the C5-chlorination of cytosine has recently been described for deoxycytidine and nucleic acids exposed to myeloperoxidase. 5-chlorocytosine appears to be a stable, albeit minority, end product, particularly at near-neutral pH. However, with suitably sensitive detection methods, 5-chlorocytosine may also prove useful as a biomarker for HOCl.

Both amino functions are involved in base pairing, so it is not surprising that HOCl^−^ induced base chlorination leads to efficient DNA denaturation. Another example of this is found in NADH where it is observed that, in the reaction with HOCl, there is a fast primary reaction and a slow secondary reaction, when there is excess HOCl, probably in this case destroying the nicotinamide ring.

**Figure 45:**
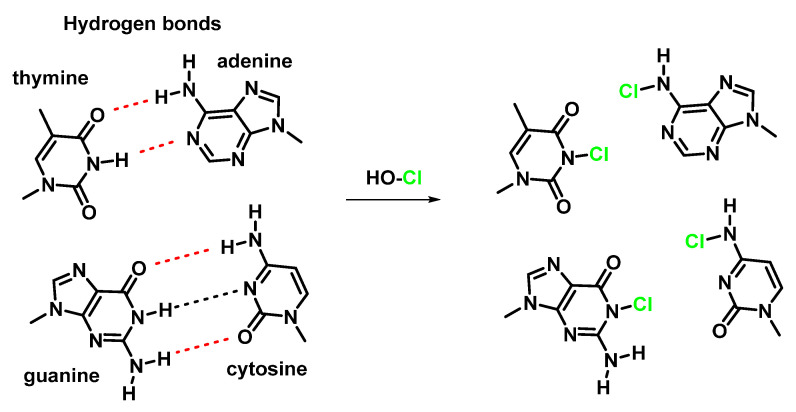
Formation of chloroamines and the breaking of the hydrogen bond.

The reaction between HOCl with DNA can lead to structural changes, and chemical modification, with the heterocyclic –NH groups R-NHR’ of guanosine and thymidine derivatives more reactive than the exocyclic NH_2_ groups R-NH_2_ of guanosine, adenosine, and cytidine derivatives [108,109,112,113]. The reaction of HOCl with these groups results in the formation of semi-stable chloramines R-NHCl and RR′NCl [114,115,116,117], which can lead to the dissociation of the DNA double strand due to the disruption of hydrogen bonds [111] and the formation of nitrogen-centred radicals [118], as shown in Figure 45.

**Figure 46:**
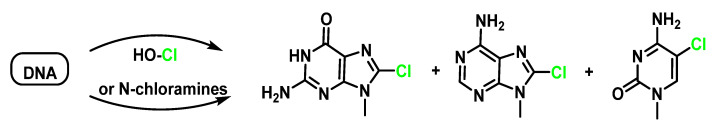
Formation of 8-chloro-2′-deoxyguanosine, 8-chloro-2′-deoxyadenosine, and 5-chloro-2′-deoxycytidine by the reaction of DNA with HOCl or *N*-chloramines.

Thymidine is the initial site of chloramine formation, from which there is a rapid transfer of chlorine atoms to the exocyclic NH_2_ groups of cytidine and adenosine. Chloramine decomposition can be accelerated by ultraviolet light, metal ions, and by thermal decomposition, giving rise to nitrogen-centred radicals derived from nucleosides and DNA. The treatment of DNA with HOCl or N-chloroamines gives 5-chloro-2′-deoxycytidine, 8-chloro-2′-deoxyadenosine and 8-chloro-2′-deoxyguanosine as end products, as shown in Figure 46.

**Figure 47:**
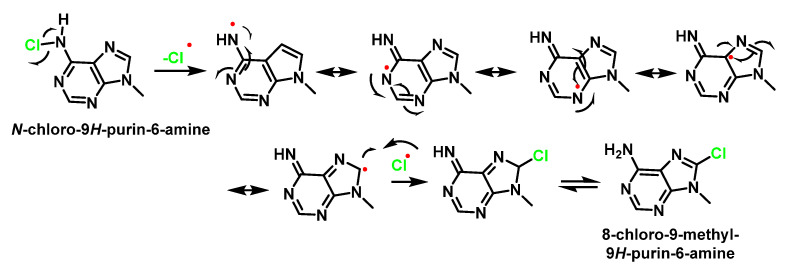
Possible mechanism of formation of 8-chloro-9-methyl-9H-purin-6-amine from the *N*-chloro-9H-purin-6-amine derivative and formation of the *N*-centred radical.

A possible mechanism of formation of 8-chloro-9-methyl-9H-purin-6-amine from the *N*-chloro-9H-purin-6-amine derivative by homolytic cleavage of the *N*–Cl bond and the formation of the nitrogen-centred radical is that of it being stabilised by the delocalisation of the odd electron through the double bonds of the two cycles, as shown in Figure 47.

Chlorination of cellular genetic material may play an important role in the development of several inflammatory cancers [35].

## 8. Reaction of HOCl with Carbohydrates

Neutrophils cause the degradation of proteoglycans, an important component of polysaccharide chains, and this process also involves MPO [119]. The main target for HOCl in carbohydrates is the nitrogen in amino sugars (D-glucosamine, D-galactosamine, D-mannosamine, etc.), in which the hydroxyl group on the 2-C atom is replaced by a primary amino group [120]. The reaction of HOCl with the free NH_2_ group of the unsubstituted glucosamine proceeds rapidly, usually even faster than with the NH_2_ group of amino acids. The formation of the dichloro amine only occurs with a large excess of HOCl.

**Figure 48:**
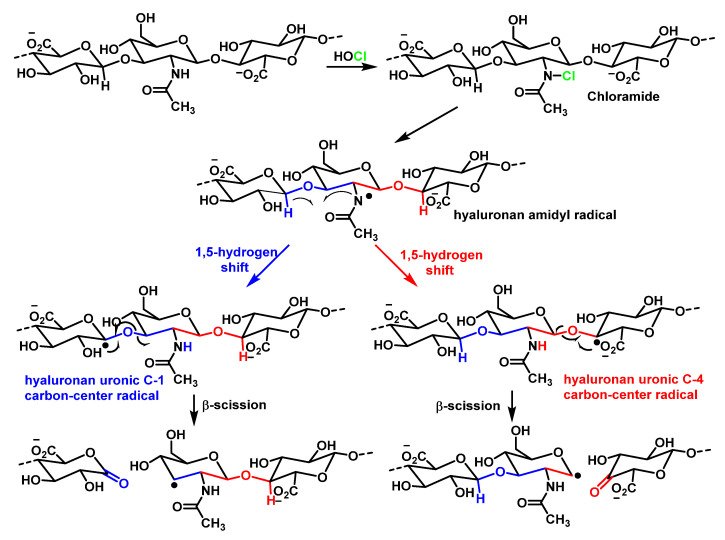
Scheme of *N*-centred amidyl-radical formation and transformation into C-centred radical with the fragmentation of the polysaccharide chain during the reaction of HOCl with the acetylamide-group of hyaluronic acid.

The reaction of HOCl with homopolysaccharides proceeds more slowly than with the corresponding monosaccharides. This can probably be explained by steric inter-action and the influence of negatively charged groups on the polysaccharides. Myeloperoxidase in the presence of H_2_O_2_ and physiological levels of chloride ion also generates chloramides from glycosaminoglycans. Chloro-derivatives of amino sugars and their substituted analogues are not stable and decompose gradually with formation of free radical intermediates [121]. One of the mechanisms of decomposition, with the example of hyaluronic acid, is shown in Figure 48.

The chloramide, which is formed in the first stage of the reaction with HOCl, is transformed into an *N*-centred amidyl radical as a result of the homolytic cleavage of *N*–Cl bonds in the presence of transition metal ions. The *N*-centred radical undergoes rearrangement and a C-centred radical is formed. This eventually leads to the fragmentation of the polysaccharide chain [122,123].

The main components of the extracellular matrix are proteoglycans, which contain many negatively charged groups on the carbohydrate side. MPO, a polycationic protein secreted by leukocytes into the extracellular space, can bind to the glycosaminoglycans of the proteoglycans by electrostatic interactions. The functioning of the enzyme in the vicinity of proteoglycans, which is accompanied by the production of HOCl, leads to the formation of carbohydrate chloramines and chloramides which decompose by a radical mechanism, as shown in Figure 44, and which is accompanied by the fragmentation of glycosaminoglycans and extracellular matrix in general [120,124].

The degradation of proteoglycans should significantly affect cell adhesion, migration, proliferation, growth, and phenotype. Free radical formation may be the cause of cellular dysfunction and the development of diseases such as atherosclerosis, rheumatoid arthritis, asthma and other diseases in which intensive MPO-dependent oxidant generation (including HOCl) is observed [125,126,127].

**Figure 49:**
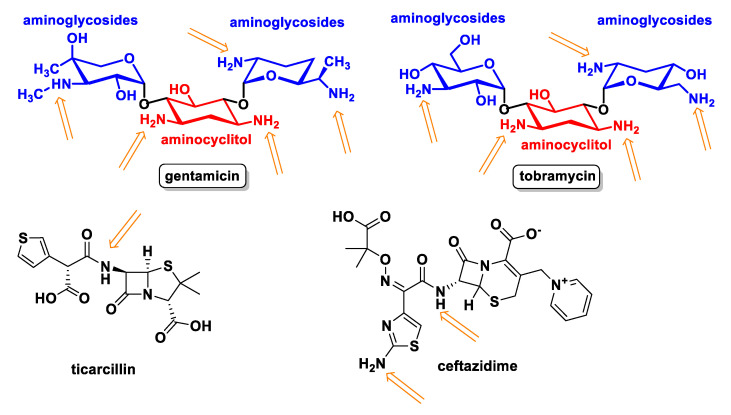
Arrows indicate possible chlorination sites with HOCl.

The aminoglycosides gentamicin and tobramycin convert HOCl into non-cytotoxic chloramines and protect lung epithelial cells against oxidative damage by the action of MPO. The aminoglycoside-aminocyclitols antimicrobials constitute one of the families of antibacterial agents with the highest activity on aerobic Gram-negative bacilli. These compounds are formed by the combination of a cyclic amino alcohol (aminocyclitol) and amino sugars (aminoglycosides) linked by glycosidic bonds. Antibiotics such as Ticarcillin (a beta-lactam antibiotic belonging to the carboxypenicillin group, one of the penicillin subgroups) and Ceftazidime (a third-generation cephalosporin), and probably other penicillin and cephalosporins, are potent HOCI scavengers and protect lung epithelial cells, specifically the sulphydryl and methionine groups, from oxidation by HOCl. These antioxidant properties of antibiotics may contribute to tissue protection in patients with cystic fibrosis [128], as shown in Figure 49.

## 9. Role of MPO in Human Diseases and Inflammation

MPO is a marker and mediator of inflammation and oxidative stress. An elevated myeloperoxidase level is associated with increased risk, prevalence, and severity, and predicts a poor prognosis in patients with cardiovascular disease. The most common CVD-related actions of MPO are: (i) generation of dysfunctional atherogenic lipoproteins; (ii) reduced NO availability; (iii) endothelial dysfunction; (iv) impaired vasoreactivity; and (v) the instability of the atherosclerotic plaque. This links the MPO levels to the pathophysiology of CVD. Thus, MPO can be considered a mediator or a tool through which inflammation promotes CVD at the molecular and cellular level. MPO can damage host tissue by generating reactive halogenating and nitrating agents [129].

MPO has a detrimental effect during chronic inflammation, and inflammation is reduced in conditions of MPO deficiency. Indeed, this has been observed in many acute and chronic inflammatory diseases. In cases of inflammatory response to non-infectious stimuli (in the absence of pathogens), several recent studies have shown an increase in the inflammatory process associated with the level of MPO.

Neutrophils and macrophages contribute to myocardial and other tissue injury resulting from post-ischemic reperfusion and inflammation [130]. These actions are mainly due to the production by these cells of HOCl/ClO^−^, superoxide ^•^O_2_^−^, and derivatives of ^•^NO [131,132,133,134].

MPO also has other catalytic activities [2]: (a) it reacts with the superoxide ^•^O_2_^−^ generating the oxo-myeloperoxidase which, in the presence of H_2_O_2_, forms “Compound II”; (b) it complexes ^•^NO and catalyses the production of the ^•^NO_2_ radical [2,133,134], using the nitrite ion as a substrate; (c) it peroxidises different oxidisable organic molecules, forming the corresponding free radicals [135,136,137]; and (d) it hydroxylates aromatic molecules [138,139].

**Figure 50:**
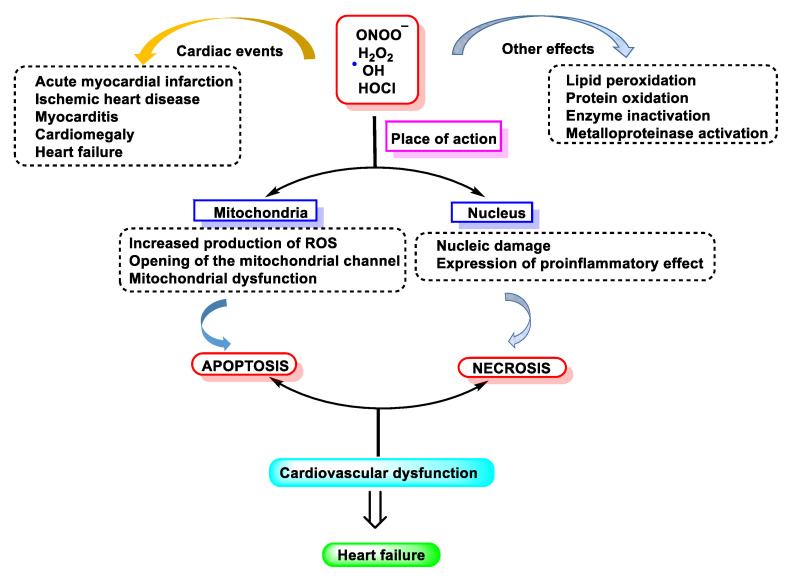
Free radical-induced damage to the cardiovascular system.

Lipoamide dehydrogenase (LADH) is a mitochondrial enzyme that is very important for energy generation in cardiac muscle. It is inhibited by oxygen free radicals, making it an optimal target for the active species generated by MPO [138,139,140,141]. These species inactivate LADH and thus may contribute to tissue damage by other oxy-radicals (Fenton reaction) as a consequence of post-ischemic reperfusion or inflammation. Compounds with thiol groups, some of which are used therapeutically, counteract the pro-oxidant action of MPO-dependent systems, as shown in Figure 50.

### 9.1. MPO in Human Diseases

In this section, we selected a number of diseases that are known to be positively related to the MPO enzyme and HOCl. This topic is more extensively reflected in Table 1, specifically in Section 9.3.

MPO is linked to many aspects of human cardiovascular disease and it is believed that this enzyme acts on both the initiation and propagation of cardiac pathologies [142]. The latter findings agree with results of many other studies that support the concept that MPO plays an important role in the pathogenesis of atherosclerosis and cardiovascular disease. Evidence supports the fact that MPO plays a very important role in the pathogenesis of atherosclerosis because it contributes to vascular dysfunction during acute inflammation by modulating the endothelial NO bioavailability [143]. A recent animal model study of ischaemia-related myocardial damage revealed increases in MPO in arrhythmogenic left ventricular remodelling, as manifested by connexin 43 rupture due to MMP-7 activation and increased post-ischaemic ventricular fibrosis [144]. Thus, MPO contributes to vascular dysfunction by virtue of its capacity to generate potent ROS and to promote the activity of matrix metalloproteinase MMPs [145]

The accumulation of LDL-derived cholesterol in the arterial wall triggers atherosclerosis, the main cause of cardiovascular disease. HDL, on the other hand, delays atherosclerosis by promoting cholesterol efflux. It has been proposed that HDL loses its cardioprotective effects in patients suffering from atherosclerosis [146]. One potential pathway involves oxidative damage by MPO; Baohai Shao et al., 2010, demonstrated that HDL from patients with cardiovascular disease contains high levels of 3-chlorotyrosine and 3-nitrotyrosine, two characteristic products of MPO [46].

MPO and HOCl^−^ modified proteins have been detected in diseased renal tissue. Mollenhauer et al., 2017, observed a significant reduction in renal function loss after the reperfusion of chemically damaged kidneys in MPO-KO mice compared to WT mice, demonstrating a contribution of MPO in the induction of organ damage after renal ischaemia-reperfusion by influencing critical factors such as neutrophil extravasation [144].

Chronic inflammation plays a key role in tumour promotion in lung cancer. Amy L. Rymaszewski et al., 2014, in mouse studies demonstrated that neutrophils are critical mediators of tumour promotion in methylcholanthrene (MCA)-initiated and butylated hydroxytoluene (BHT)-promoted lung carcinogenesis and subsequently, they investigated the role of neutrophil MPO activity in the inflammation-promoted model, observing increased protein levels and MPO activity in the lungs of mice-administered BHT. An MPO inhibitor reduced tumour burden.

Rotem Volkman et al., 2019, reviewed the body of evidence linking neutrophil-derived MPO in the pathogenesis of Alzheimer’s disease (AD), verifying this role in an animal model. They reproduced haematological chimerism in the 5XFAD mouse model of AD with MPO-deficient mice, resulting in 5XFAD with haematological MPO deficiency (5XFAD-MPO KO). Behavioural examinations of 5XFAD-MPO KO mice showed a significantly superior performance in spatial learning and memory, associative learning and anxiety/risk assessment behaviour compared to 5XFAD mice transplanted with WT cells (5XFAD-WT). Immunohistochemical and hippocampal mRNA expression analyses showed significantly reduced levels of inflammatory mediators in 5XFAD-MPO KO mice, with no apparent difference in the number of amyloid-β plaques. In addition, immunoblotting and mRNA analyses showed significantly reduced levels of APOE in 5XFAD-MPO KO mice.

Taken together, their analyses indicate a substantial involvement of neutrophil-derived MPO in the pathogenesis of the 5XFAD model of AD and suggest that MPO is a potential therapeutic target in AD.

In recent years, a significant amount of evidence has implicated a role of MPO in the pathogenesis of atherosclerosis. MPO is an enzymatic source of eicosanoids and bioactive lipids and generates atherogenic forms of low- and high-density lipoproteins. These factors demonstrate that increased systemic levels of MPO and its oxidation products predict increased cardiovascular risk. Consequently, interest has focused on the potential of MPO for the development of new mechanisms to analyse its presence as risk markers, as well as therapies to prevent cardiovascular events. Rachel J. Roth Flach et al., 2019, examined the role of MPO inhibitors in the treatment of heart failure and acute coronary syndrome in humans. The results obtained in their study suggest that MPO inhibition does not alter the atherosclerotic plaque area or leukocyte uptake, but rather alters the inflammatory tone of atherosclerotic lesions; therefore, MPO inhibition has utility in promoting atherosclerotic lesion stabilisation and preventing atherosclerotic plaque rupture [147].

### 9.2. MPO and Organ Inflammation

MPO can damage the host tissue by generating reactive halogenating and nitrating agents. Lower levels of 3-chlorotyrosine, 3-bromotyrosine, 3-nitrotyrosine, and protein carbamylation are observed at sites of inflammation in MPO-KO mice compared to WT mice. Therefore, since MPO has a detrimental effect during chronic inflammation, it is to be expected that inflammation is reduced under conditions of MPO deficiency. Indeed, this has been observed in many acute and chronic inflammatory diseases [148].

Recent observations extend this perspective and deeply implicate MPO in the regulation of cellular homeostasis, playing a central role in the initiation and propagation of acute and chronic vascular inflammatory disease. Thus, low levels of HOCl interfere with intracellular signalling, as MPO-dependent lipoprotein oxidation modulates its affinity for macrophages and the vascular wall. Simultaneously, MPO-mediated endothelial NO depletion impairs vasodilation and nitrotyrosine (NO_2_Tyr) formation in the vascular wall may affect the structure and function of matrix proteins [149].

The development of imaging techniques to accurately identify MPO localisation and molecular targets of HOCl in vivo is an important advance. Until recently, the involvement of MPO in inflammatory disease has been inferred by its presence, together with the detection of HOCl biomarkers, in biological fluids or diseased tissues. These results provide valuable information regarding the cell types responsible for MPO release in vivo, along with new insight into potential therapeutic opportunities [16].

### 9.3. Summary Table of the Relationship between MPO and Different Diseases

As follows, Table 2 summarises the relationship between MPO and disease (including recent research findings), citing diseases and references.

**Figure 51:**
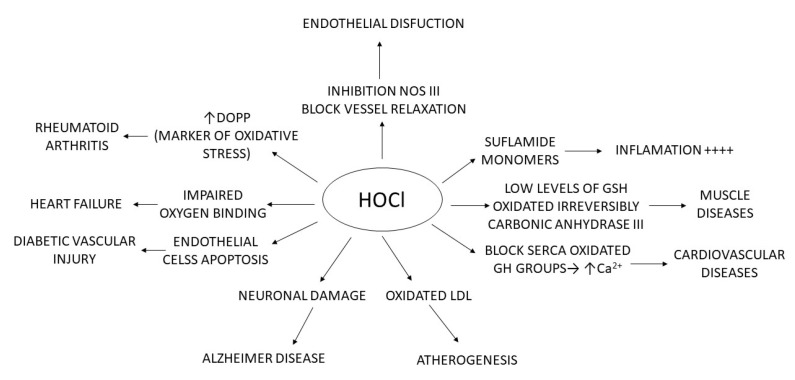
Relationship of HOCl with different pathologies.

Figure 51, below, summarises what has been explained throughout the manuscript: chlorinated species have mainly been related to pathologies where the cause is linked to molecular alterations and their effect on inflammatory processes, tissue damage, damage to genetic material, apoptosis, etc.

### 9.4. Myeloperoxidase as a Disease Biomarker

The antibacterial activity of MPO, through the production of HOCl and its controlled release at the site of infection, is of vital importance for its activity to be effective. Its uncontrolled expression overstates inflammation and can lead to tissue damage, even if inflammation does not occur. Several tissue lesions and the pathogenesis of diseases such as rheumatoid arthritis, cardiovascular and liver diseases, diabetes and cancer are linked to MPO-derived HOCl. Therefore, increased MPO activity is a good diagnostic tool for biomarkers of inflammation and oxidative stress [6].

Coordination between several biochemical pathways, including neutrophil activation, ^•^O_2_^−^ production by NADPH oxidase, and MPO release by exocytosis, leads to clearance of bacterial infection [57], as invading bacteria induce increased H_2_O_2_ production by the enzyme superoxide dismutase SOD, whereby MPO produces HOCl. Both products, H_2_O_2_ and HOCl, are particularly toxic to invading bacteria. This biochemical phenomenon is called the respiratory burst [210]. When bacterial infection occurs, one of the important mediators of this cascade is formylated peptide, which also acts as a chemoattractant, activating neutrophils via the formylated peptide receptor fPR, a G protein-coupled receptor [211]. The release of H_2_O_2_ oxidises various substrates, such as halides (Cl^−^, Br^−^), and pseudohalides (thiocyanate SCN^—^) [8,212]. These oxidant species, under normal physiological circumstances, are toxic to several micro-organisms and play an important role in the immune system. Their excessive or deregulated production of oxidants can cause damage to host cells and lead to various diseases [213,214]. The polycationic character of MPO allows it to bind to negatively charged surfaces of pathogens and causes the destruction of their cell membrane, inducing lysis of the bacteria. This enzyme can also bind to other cell surfaces, such as epithelial cells, macrophages, fibroblasts, endothelial cells, platelets, neutrophils, low-density lipoproteins (LDL), and very low-density lipoproteins (VLDL) [215,216].

### 9.5. Measurement of MPO Activity

Currently, there is considerable interest in developing “biomarkers” of MPO activity that can be applied to humans. These involve measuring the end products of oxidative damage in different classes of biomolecules or directly determining the production of HOCl. Different types of equipment are currently available on the market. The most common method of measuring MPO is through enzyme-linked immunosorbent assay ELISA kits. Among the different devices, the following are worth mentioning:

Arigo Biolaboratories Corporation (Hsinchu City, Taiwan), developed a MPO/Myeloperoxidase Activity Assay Kit (Colorimetric) that can be used to measure myeloperoxidase activity in whole neutrophils, neutrophil lysates, tissue homogenates, and plasma (EDTA). This colorimetric device bases its principle of operation on the fact that HOCl rapidly reacts with taurine to produce a stable taurine chloramine product. This step neutralises HOCl, which would otherwise accumulate and inactivate MPO. A stop solution containing catalase is added to stop the catalysis of MPO, removing the hydrogen peroxide. Finally, taurine chloramine reacts with the yellow chromogenic probe TNB, with a decrease in colour indicating increased MPO activity. The concentration of MPO in the samples is then determined by comparing the absorbance of the samples at 405–412 nm with the standard curve.

Abcam, at the Cambridge Biomedical Campus, Cambridge, UK, markets a colorimetric kit with similar characteristics to that described above, notably that it can be used to detect MPO as low as 0.05 mU per well.

Cayman Chemical Company, in Ann Arbor, MI, USA, markets a complete assay for the isolation of neutrophils and the measurement of MPO activity that analyses the release of MPO by activated phagocytes and uses TMB as a chromogenic substrate for MPO, including a specific inhibitor of MPO function to verify specificity. This also includes the reagents necessary to isolate neutrophils from human whole blood.

Celltechgen Laboratory, in Houston, TX, USA, is a state-of-the-art medical testing laboratory service that provides a complete range of tests for the diagnosis, screening, or evaluation of diseases and health conditions and markets an MPO Peroxidation Activity Assay Kit, suitable for use as a high-throughput MPO activity assay. The assay kit oxidises a substrate to generate fluorescence (Ex/Em = 571/585 nm), directly proportional to total peroxidase activity in the sample. The assay is high-throughput adaptable and can detect less than 2 µU of MPO activity. This kit can be used to detect MPO activity as low as 0.5 µU per well.

### 9.6. Inhibitors of MPO

The microbicidal activity of the MPO enzyme is due to its ability to oxidise halide or pseudohalide ions (X = Cl^−^, Br^−^, I^−^ and SCN^−^) producing the respective HOX acids. During the phagocytosis of pathogens, MPO is released by azurophilic granules into phagolysosomes but can also be discharged outside phagocytes. Tissue damage that occurs during inflammation is largely due to MPO-derived oxidants. As referenced in previous chapters, this enzyme is a key factor in several conditions, including cardiovascular, inflammatory, neurodegenerative, renal, immune-mediated, and neurodegenerative diseases. Therefore, MPO is an attractive target for therapeutic intervention in the prophylaxis of the aforementioned disorders. The only negative effect that can be expected from MPO inhibitors is a decrease in neutrophil activity against pathogens. MPO is an immunological enzyme that acts in neutrophils. However, MPO is located in azurophil granules, which protect this enzyme from changes in the extracellular environment. Therefore, it is believed that the effect of inhibitors on MPO activity can be easily attenuated by exclusively focusing on extracellular enzymes that are not critical for pathogen eradication but rather are involved in host damage.

Relatively polar MPO inhibitors cannot penetrate neutrophils and are thought to inhibit extracellular enzymes exclusively. The structure and reaction mechanism of MPO is known, allowing a rational strategy for the development of specific inhibitors, with the intention of preserving its activity against bacteria, but hindering its pathophysiological persistent activation during the course of the diseases [217].

There are three approaches to discover and develop such inhibitors:

The first approach is to prepare tiny compounds with a reduction potential of 0.97 V E°′ (A-/AH) 1.35 V. MPO compound I can easily oxidise these molecules, leading to the inactive state MPO compound II. At the same time, they cannot degrade the MPO compound II and cause this inactive form of MPO to build up. The main problem with these inhibitors is that when they are used in living organisms, several biomolecules can act as substrates for MPO and degrade the MPO compound II to restore the original enzyme. As a result, many inhibitors lose their efficacy in vivo.

The second approach focuses on tiny molecules that bind with high affinity to the active site of MPO. These chemicals are designed to strongly interact with the active site residues of the enzyme. The key amino acid that forms a salt bridge or hydrogen bond with the inhibitor is Glu102. In addition, to convert the MPO compound I into the MPO compound II, the inhibitor must have a reduction potential E°′ (A-/AH) of less than 1.35 V. When this type of molecule induces the inactive state of the enzyme, it maintains its contacts with the active site of MPO and prevents additional substrates from entering the active site, leading to the accumulation of MPO compound II by competitive inhibition. Several effective MPO inhibitors, such as aminoalkyl-indole compounds and aryl hydroxamic acid derivatives [47,48,49,50], were prepared using this strategy.

The third approach is to prepare tiny molecules that form covalent bonds after oxidation by MPO and have a relatively high affinity for the enzyme. These compounds are irreversible MPO inhibitors that act by degrading the heme group. Inhibitors that rely on overriding this mechanism are irreversible and form a strong covalent bond with the Fe of the haem centre, blocking the access of H_2_O_2_ to the active site, and thus inactivating the enzyme. The second mechanism is based on inducing competition between the inhibitor compound and the enzyme substrate. In this case, the inhibitor either forms a complex with MPO preventing further cycling or acts as a substrate for MPO by forming and accumulating compound II. An alternative approach based on the design of HOCl scavenging compounds can be considered in order to avoid the induced oxidative damage. However, this mechanism will not prevent the peroxidation cycle and the formation of superoxide and hydroxyl radical, which are involved in oxidative stress and tissue injury. Due to the complexity of the MPO catalytic mechanism, the search for an effective inhibitor is still under development [218].

Lifestyle factors are important drivers of chronic diseases such as cardiovascular syndrome, with inflammation being a key factor. MPO is an inflammatory enzyme associated with obesity, hypertension, and heart failure, so its attenuation could have protective effects on multiple organs. Arnold Piek et al., 2019, tested the effects of a novel oral MPO inhibitor AZM198 in an obese/hypertensive mouse model with a cardiac phenotype Treated animals showed therapeutic AZM198 levels of 2.1 µM, corresponding to a 95% inhibition of MPO. AZM198 reduced elevated circulating MPO levels in HFD/AngII mice to normal values. Independently of food intake, body weight gain and fat accumulation were attenuated, along with reduction in visceral adipose tissue (VAT) inflammation and the attenuation of the severity of non-alcoholic steatohepatitis [219].

Most peroxidase enzymes are inhibited by benzoic acid hydrazide (BAH)-containing compounds, but the inhibition mechanism by BAH compounds is unknown. Jiansheng Huang et al., 2015, reported that the MPO inhibition by BAH and 4-(trifluoromethyl)-BAH due to hydrolysis of the ester bond between the MPO heavy chain glutamate 242 ((HC)Glu(242)) residue and the heme pyrrole A ring. In their manuscript, they provide evidence that the destruction of the heme ring does not occur by heme prosthetic group tracking and provides indications that the mechanism of hydrolysis follows a potential attack of the carbonyl of (HC)Glu(242), leading to a rearrangement that causes the release of the vinyl-sulphonium bond between (HC)Met(243) and the pyrrole A-ring [220].

Clinical studies have been only conducted on AZD5904, AZD3241, AZD4831, and PF06282999. The first three chemicals are thioxanthine derivatives, while the fourth is a thiopyrimidinone. The common mechanism of action of these drugs is a thioether bridge between the heme group of the enzyme and the oxidised thioxantine or thiopyrimidinone. Thioxanthins are compounds known as MPO inhibitors and derivatives with the 2-thioxanthin group show the highest activity. Thioxanthine is oxidised by compound I, forming a highly reactive free radical. This radical readily transfers electrons to the heme group of compound II and forms a covalent bond across the sulphur atom to one of the heme’s pyrrole rings [221].

Several naturally occurring compounds possess inhibitory activities against MPO, including polyphenols, melatonin, and flavonoids. Seeds and aerial parts of *Peganum harmala* L. are widely used in Algeria as anti-inflammatory remedies. Sihem Bensalem et al. evaluated in their study the alkaloids and pure β-carboline compounds present, as well as their possible anti-inflammatory and MPO inhibitory action, concluding that the total alkaloids from seeds and aerial parts strongly inhibited MPO at 20 µg/mL (97 ± 5% and 43 ± 4%, respectively) while, at the same concentration, those from the roots showed very low inhibition (15 ± 6%) [222].

On the other hand, ceruloplasmin is a plasma protein produced by activated hepatocytes and macrophages and is involved in the physiological clearance and inactivation of MPO [223]. In addition to these, some naturally occurring compounds, such as polyphenols, with pronounced antioxidant and anti-inflammatory characteristics, have inhibitory activities against MPO. These compounds include ferulic acid, caffeic acid, resveratrol, chalcones, and gallic acid. [224,225]. Yuko Shiba et al., 2008, examined the MPO inhibitory effects of dietary flavonoids, using a combination of biological assays and theoretical computational studies. Quercetin and the plasma metabolites inhibited the formation of dityrosine catalysed by the MPO enzyme and HL-60 cells in a dose-dependent manner [226].

## 10. Conclusions

HOCl is the most important reactive form of chlorine formed in living organisms in the MPO halogenation cycle. HOCl biologically falls into a group of small molecules known as reactive oxygen species (ROS) synthesised by the cells of the immune system (neutrophils and macrophages). HOCl-dependent reactions play a dual role: on the one hand, it performs a bactericidal function against infections, and on the other hand, it can cause damage to supramolecular structures and cells of the host organism. In this review, HOCl is presented as a powerful antimicrobial oxidant, capable of modifying DNA, lipids, and lipoproteins, rapidly reacting with nucleophiles such as the sulphur atom present in thiols and thioethers (cysteine and methionine). Modification of amines and amides by reaction with HOCl generate chloramines and chloramides, respectively, in virtually all organic biological compounds, which could have serious cellular implications. It is also important to note that the oxidation of tyrosine residues by HOCl leads to the formation of 3-chlorotyrosine, which is considered a marker of MPO activity and halogenative stress.

## Figures and Tables

**Table 1:** Second-order rate constant for backbone amides varies over several orders of magnitude, depending on the environment. This value is a maxima estimate based on studies with cyclic dipeptides.

Residue	K_2_ M^−1^ s^−1^	Residue	K_2_ M^−1^ s^−1^
Met	3.8 × 10^7^	Lys	5.0 × 10^3^
Cys	3.0 × 10^7^	Tyr	44
Cistine	1.6 × 10^5^	Arg	26
His	1.0 × 10^5^	Backbone amides	<10
α-amino	1.0 × 10^5^	Asn	0.03
Trp	1.1 × 10^4^	Gln	0.03

**Table 2:** Occurrence of MPO in various diseases.

Disease Classification	Disease and References
Autoimmune Disease	Inflammatory bowel disease/colitis [150,151,152] Rheumatoid arthritis [153,154] Systemic lupus erythematosus [155,156,157]
Neuronal Pathology	Alzheimer’s disease [158,159,160] Multiple sclerosis [161,162,163] Neurodegenerative disease [164,165,166] Parkinson’s disease [167,168] Stroke [169,170,171]
Cardiovascular Pathology	Atrial fibrillation [172,173] Cardiovascular disease/atherosclerosis [174,175,176] Hypertension [177,178,179] Myocardial infarction [180,181,182] Vascular dysfunction [183,184,185] Asthma [186,187,188]
Pulmonary Pathology	Chronic obstructive pulmonary disease [189,190,191] Cystic fibrosis [192,193,194]
Miscellaneous	Ageing [195,196,197] Cancer [198,199,200] Chronic kidney disease [201,202,203] Inflammation [16,129,204] Lipoprotein modification [151,182,183] Metabolic syndrome/obesity [205,206,207] Type 2 diabetes [207,208,209]

## Data Availability

Not applicable.
